# Shi Wei Ru Xiang pill alleviates acute gouty arthritis through suppressing NLRP3 inflammasome activation

**DOI:** 10.3389/fphar.2025.1595578

**Published:** 2025-06-25

**Authors:** Na Wang, Puchen Zhao, Qin Yin, Lizi Li, Haiqi Xu, Can Yang, Yanbei Tu, Yanfang Li

**Affiliations:** ^1^ School of Chemical Engineering, Sichuan University, Chengdu, Sichuan, China; ^2^ School of Pharmacy, Jiangsu University, Zhenjiang, Jiangsu, China

**Keywords:** Shi Wei Ru Xiang pill, NLRP3 inflammasome, acute gouty arthritis, Tibetan medicine, monosodium urate

## Abstract

**Introduction:**

Shi Wei Ru Xiang pill (SWR) is commonly utilized in Tibetan medicine as a therapeutic intervention for “Huang-shui disease” and has been clinically validated as an effective treatment for acute gouty arthritis (AGA). Nevertheless, the underlying mechanisms of action and the active components of SWR in combating AGA remain unclear.

**Methods:**

In this study, the effects of SWR and its active components on AGA and NLRP3 inflammasome activation were investigated in monosodium urate (MSU)-induced AGA rats and lipopolysaccharide/nigericin-induced THP-1 cells. The chemical profile of SWR was characterized using UPLC-Q-Exactive Orbitrap MS.

**Results:**

The results indicated that SWR effectively suppressed pyroptosis, caspase-1 activity, and IL-1β production in THP-1 cells. Furthermore, SWR significantly suppressed NLRP3 inflammasome activation and attenuated ankle swelling in a rat AGA model. Specifically, SWR affected the priming and assembly phases to inhibit NLRP3 inflammasome activation. Surprisingly, SWR showed good liver and renal protective effects in AGA rats. A total of 58 compounds were identified in SWR by UPLC-MS analysis. Further pharmacological studies demonstrated that the phenolic compounds serve as active compounds responsible for the inhibition of inflammasome activation, including dehydrocostus lactone, gallic acid, 4-hydroxybenzoic acid.

**Discussion:**

This is the first study to comprehensively elucidate the therapeutic effects and underlying mechanisms of SWR against AGA by inhibiting NLRP3 inflammasome activation. This study strongly indicated that SWR could serve as a promising anti-inflammatory medicine with an acceptable safety profile for the treatment of AGA and other inflammatory disorders linked to NLRP3 inflammasome activation.

## Graphical Abstract

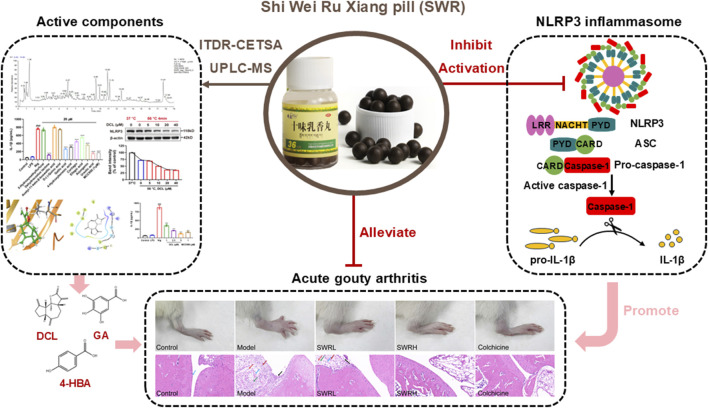

## Highlights

• Integrated various approaches first revealed that the therapeutic effects and underlying mechanisms of SWR against AGA through inhibiting NLRP3 inflammasome activation.
• UPLC-MS analysis identified 58 compounds of SWR and revealed its active compounds.
• The binding of the active compounds of SWR to the NLRP3 protein was verified through molecular docking and isothermal dose-response cellular thermal shift assay (ITDR-CETSA).

## 1 Introduction

Acute gouty arthritis (AGA) is a prevalent inflammatory disease caused by the accumulation of monosodium urate (MSU) crystals in joints (Wang Y. et al., 2023). This disease is marked by significant swelling and intense discomfort in the lower extremity joints, and can eventually lead to deformity if left untreated (Keyβer, 2020). Recently, which has become a growing serious public health concern. In China, the prevalence of AGA in populations aged >40 years is approximately 46.3%, increasing annually (Filardo et al., 2016). It should be stressed that the therapeutic options for treating AGA are limited to colchicine, glucocorticoids, and nonsteroidal anti-inflammatory drugs (NSAIDs) (FitzGerald et al., 2020; Keller and Mandell, 2021). In addition, the clinical utilization of these medications is hindered by their respective hepatorenal toxicity, gastrointestinal adverse effects, and other complications (Ragab et al., 2017). Therefore, there is a pressing need to develop safer, more potent, and inexpensive therapeutic agents for patients with AGA.

AGA pathogenesis is characterized by an inflammatory response elicited by MSU crystal interactions with the local microenvironment, resulting in multiple pathological changes including cellular destruction, tissue injury, or functional impairment. It is commonly accepted that these changes are primarily associated with the overexpression of pro-inflammatory cytokines (Zhao et al., 2022) and the activation of nucleotide-oligomerization domain-like receptor (NLR) family pyrin domain-containing 3 (NLRP3) inflammasome (Martinon et al., 2006). Among these inflammatory cytokines, interleukin-1β (IL-1β) serves as a central driver of joint inflammation associated with gout by facilitating vasodilatation and promoting the recruitment of monocytes and the infiltration of neutrophils to areas where MSU crystals are deposited (Dalbeth et al., 2021). Continuous IL-1β secretion further contributes to the production of enzymes responsible for matrix degradation, which ultimately destroys the cartilage and bone (Schlesinger and Thiele, 2010).

NLRP3 inflammasome is a multiprotein complex that consists of the innate immune sensor NLRP3, adaptor protein apoptosis-associated speck-like protein containing a caspase activation and recruitment domain (ASC), and the effector protein cysteinyl aspartate-specific proteinase-1 (caspase-1) (Lamkanfi and Dixit, 2014). As an important pattern recognition receptor in innate immunity, it is triggered by pathogen-associated molecular patterns (PAMPs) or damage-associated molecular patterns (Sharif et al., 2019; Swanson et al., 2019). The persistent and excessive NLRP3 inflammasome activation is well-established to be closely associated with the gout progression. Its activation facilitates the release of pro-inflammatory cytokine IL-1β, a key pro-inflammatory cytokine that mediates gout. Furthermore, its activation initiates pyroptosis, a form of programmed inflammatory cell death (Jiang et al., 2020; Liu et al., 2016). Therefore, inhibiting NLRP3 inflammasome activation could be of great significance for treating AGA. Recently, IL-1β inhibitors and biological drugs blocking NLRP3 activity have been proven to play an increasingly important role (Jena et al., 2021). Whereas, the high price, risk of severe infections, and potential to induce an exaggerated immune response *in vivo* have limited their clinical applications (Bettiol et al., 2019). Therefore, it is still attractive to explore novel therapeutic options for AGA by targeting NLRP3 inflammasome.

Owing to the unique geographic environment and regional lifestyle, the incidence rate of AGA in Tibet is significantly higher than that in other regions (Sun et al., 2018). Interestingly, for the treatment of AGA, Tibetan medicine has its characteristics and advantages. There are 22 types of commonly used patent medicines, the most frequently used of which are Twenty-Five Wei’ er Tea Pills, Gouty Decoction, and Shi Wei Ru Xiang Pill (SWR) (La et al., 2021). As a commonly used preparation for treating “Huang-shui disease” with a long history of clinical experience in Tibetan medicine, the prescription of SWR comes from the traditional Tibetan medical classics “Four Medical Tantras” (四部医典). Recent studies have demonstrated that SWR significantly inhibited collagen-induced arthritis in rats by suppressing MAPK and STAT3 signaling pathways (Xiong et al., 2023) and ameliorated hyperuricemia in mice by regulating the NF-κB and MAPK pathways (Li Q. et al., 2022). Although these previous studies have revealed the therapeutic effects and mechanisms of SWR on rheumatoid arthritis and hyperuricemia, the comprehensive therapeutic effects of SWR on AGA and underlying mechanisms remain largely unknown. In addition, the regulatory effects of SWR on NLRP3 inflammasome activation have not been reported. Notably, there are few reports concerning the active components of SWR in AGA treatment, which hinders its clinical application and further development. Therefore, it is necessary to reveal the therapeutic effect of SWR on AGA from a holistic perspective and explore the mechanism of action of SWR with a focus on the regulation of NLRP3 inflammasome activation.

In view of above perspective, in current study, a lipopolysaccharide (LPS)/Nigericin (Nig) induced inflammatory cell model in THP-1 cells and an MSU-induced AGA model in rats was adapted in the hope of further revealing its anti-inflammatory effect on AGA from a holistic perspective and mechanism of action with a focus on the regulation of NLRP3 inflammasome activation.

## 2 Materials and methods

### 2.1 The material and preparation of SWR samples

Shi Wei Ru Xiang Pill (SWR, Lot number 221045) was purchased from Tibet Qizheng Tibetan Medicine Co., Ltd. (Lhasa, Tibet, China) and deposited at the laboratory of the School of Chemical Engineering, Sichuan University (Sichuan, China). Its composition is presented in Table 1. According to preparation process included by the criteria of National Drug standards (WS3-BC-0211-95), the ten raw materials, except for Zhaxungao, are crushed into fine powder, sieved and mixed well, then mixed with Zhaxungao and an appropriate amount of water to form pills, the finished product is obtained after drying.

**TABLE 1:** The composition of Shi Wei Ru Xiang Pill (SWR).

NO.	Chinese name	Latin name	Source species	Parts used	Weight (g)
1	Hezi	*Chebulae Fructus*	*Terminalia chebular* Retz.	Fruit	150
2	Yuganzi	*Phyllanthi Fructus*	*Phyllanthus emblica* L.	Fruit	120
3	Ruxiang	*Olibanum*	*Boswellia carterii* Birdw.	Resin	100
4	Kuanjinteng	*Caulis Tinosporae Sinensis*	*Tinospora sinensis* (Lour.) Merr.	Stem	100
5	Maohezi	*Terminaliae Belliricae Fructus*	*Terminalia billirica* (Gaertn.) Roxb.	Fruit	100
6	Muxiang	*Aucklandiae Radix*	*Aucklandia lappa* Decne.	Root	85
7	Juemingzi	*Cassiae Semen*	*Cassia obtusifolia* L.	Seed	80
8	Huangkuizi	*Abelmoschus moschatus Medicus*	*Abelmoschus manihot* (L.) Medic.	Seed	80
9	Baxiaga	*Adhatoda vasica Nees*	*Veronica eriogyne* H.Wink1.	Stem	80
10	Zhaxungao	*Brag-zhun*	Shilajit	Mineral	50

For the preparation of SWR samples, the powders of SWR pills (5g) were soaked in 10 mL of dimethyl sulfoxide and extracted in an ultrasonic bath at room temperature (30 min × 3). The obtained extract was filtered using a 0.22 μm filter, which was stored at 4°C for further analysis.

### 2.2 Reagents and antibodies

Monosodium urate (MSU) was acquired from Aladdin Reagent Co., Ltd. (Shanghai, China). Phorbol 12-myristate 13-acetate (PMA) and lipopolysaccharide (LPS) were purchased from Sigma-Aldrich Chemicals Co., Ltd. (St. Louis, USA). Adenosine triphosphate (ATP), Disuccinimidyl suberate (DSS), Propidium Iodide (PI) and NP-40 lysis buffer were purchased from Meilunbio (Dalian, China). Nigericin (Nig) was acquired from Invivogen (Toulouse, France). MCC950 was acquired from Med Chem Express (Trenton, USA). Hochest 33342 and Caspase 1 Activity Assay Kit were purchased from Beyotime Biotechnology (Shanghai, China). Colchicine and uric acid were purchased from Yuanye Bio-Technology Co., Ltd. (Shanghai, China). Fetal bovine serum (FBS) was purchased from Gibco (10270-106, Brazil). CytoTox 96^®^ Non-Radioactive Cytotoxicity Assay was acquired from Promega (Madison, USA). Human IL-1β ELISA kit, human TNF-α ELISA kit, rat IL-1β ELISA kit and rat TNF-α ELISA kit were supplied by 4A Biotech (Suzhou, China). Assay kits of UA, blood urea nitrogen (BUN), creatinine (Cr), alanine amino transferase (ALT), aspartate amino transferase (AST) were offered by Nanjing Jian Cheng Bioengineering Institute (Nanjing, China). Antibodies against GAPDH and β-actin were purchased from Abways (Shanghai, China). Anti-ASC antibody and anti-rat caspase-1 antibody was supplied by Santa Cruz Biotechnology (Dallas, USA). Anti-NLRP3 antibody was supplied by Abcam (Cambridge, UK). Anti-human caspase-1 antibody was purchased from Adipogen (San Diego, USA). Anti-IL-1β antibody was purchased from Affinity (Changzhou, China). Anti-GSDMD antibody was supplied by Huabio (Hangzhou, China). All other reagents and solvents were analytically pure and the ultra-pure water was used throughout the experiment.

Huamn THP-1 cells were acquired from Procell Life Science & Technology Co. Ltd. (Wuhan, China). The cells were cultured in RPMI 1640 medium supplemented with 10% fetal bovine serum (Gibco, USA), 100 μg/mL streptomycin, and 100 units/mL penicillin in a humidified atmosphere of 95% air and 5% CO_2_ at 37 °C.

### 2.3 UPLC-Q-Orbitrap-MS analysis of SWR

UPLC-MS analysis was conducted using a Vanquish Liquid chromatography/Q Exactive Plus Orbitrap mass spectrometer. SWR (10 mg/mL) was prepared by dissolving it in MeOH, straining using a 0.22 μm filter, and analyzed using UPLC. The separation process utilized a HYPERSIL GOLD VANQUISH C_18_ column (2.1 mm × 100 mm, 1.9 μm) using a gradient elution comprising 0.1% aqueous formic acid (A) and acetonitrile (B) as follows: initially 2% B (0–3 min), increased to 30% B (3–5 min), raised to 70% B (5–14 min), elevated to 98% B (14–16 min), and decreased to 2% B (16–19 min). The column was maintained at 30°C with a 0.35 mL/min flow rate and injection volume of 2 µL. Mass spectrometry was performed using a Q Exactive Plus mass spectrometer fitted with an electrospray ionization source (Thermo Fisher Scientific, USA). Mass spectra were acquired across a range of 100.0–1,500.0 *m/z*, with chromatographic data collected in negative and positive ionization modes. Data processing and analysis were performed using the Xcalibur software.

### 2.4 Inflammasome stimulation

To induce NLRP3 inflammasome activation, THP-1 cells were cultured in 48-well plates and subsequently treated with 100 ng/mL PMA for 24 h to get differentiated cells. Following this incubation period, the cells were triggered with 1 μg/mL LPS for 3 h. Subsequently, the cells were incubated for an additional 1 h with 50 and 100 μg/mL SWR. The cells were then stimulated with 10 μM Nig for 40 min, 5 mM ATP for 1 h, 300 μg/mL aluminum salts (Alum) for 6 h, or 500 μg/mL MSU.

### 2.5 Cell viability assay

The cytotoxicity of SWR was determined using the MTT assay. The experimental procedures were conducted following the previously described method (Du et al., 2024).

### 2.6 Enzyme-linked immunosorbent assay (ELISA)

After stimulation, the cell culture supernatant was centrifuged to remove cellular debris. Rat blood samples were collected via the abdominal aorta and centrifuged for 15 min. The joint cavity of rats was thoroughly irrigated with sterile saline and the resulting lavage fluid was collected and centrifuged. The concentrations of IL-1β and TNF-α in the culture medium supernatants, rat serum, and joint cavities were quantified using commercial ELISA kits.

### 2.7 Cell pyroptosis assay

Cells were seeded at a density of 1 × 10^6^/mL in 24-well plates. Differentiated THP-1 cells were primed for 3 h with LPS (1 μg/mL). Subsequently, cells were treated with SWR (50 and 100 μg/mL) for an additional hour, followed by stimulation with Nig (10 μM) for 40 min. The cell supernatant was discarded, and the cells were washed with PBS for three times. Propidium iodide (PI) (5 μg/mL) and Hoechst 33342 (5 μg/mL) were applied for 20 min and were then washed with PBS. Images were acquired using an ECLIPSE Ts2R/FL inverted microscope (Nikon, Japan).

### 2.8 Lactate dehydrogenase (LDH) assay

LPS-primed THP-1 cells were seeded at a density of 5 × 10^5^/mL in 48-well plates. Cells were subsequently treated with Nig in the presence or absence of the SWR (50 and 100 μg/mL). Culture supernatants were collected and analyzed using an LDH cytotoxicity assay kit.

### 2.9 Measurement of soluble proteins p17 and p20

After inflammasome stimulation, the culture media were collected, 20% (v/v) trichloroacetic acid (TCA) was added, and the samples were incubated overnight at −20°C. The samples were centrifuged and the resulting pellets were washed three times with cold acetone. Finally, the pellets were resuspended in 1× loading buffer, and the concentrations of p17 and p20 were determined using Western blot analysis.

### 2.10 Western blot analysis

After inflammasome stimulation, the cells were lysed on ice with RIPA lysis for 30 min buffer and subsequently centrifuged. Synovial tissues from the rats were homogenized in RIPA lysis buffer. Total protein was recovered by centrifugation at 13,000 rpm for 15 min at 4 °C. The protein concentration was quantified using a BCA kit (Beyotime, China), and 5× loading buffer was added to the supernatant, followed by heating at 100°C for 10 min. Subsequent experiments were conducted using previously established methods (Du et al., 2024).

### 2.11 ASC oligomerization assay

The cells were lysed using NP-40 for 30 min. The resulting lysates were centrifuged and the pellets (NP-40 insoluble fraction) were resuspended in disuccinimidyl suberate (2 mM) and incubated for 30 min. Following incubation, the resuspended pellets were centrifuged and the cross-linked pellets were resuspended in 1× loading buffer for WB analysis.

### 2.12 Immunofluorescence

After inflammasome stimulation, cells were fixed with ice-cold methanol for 20 min. After blocking in 2.5% bovine serum albumin for 1 h at 25°C, the cells were incubated overnight with ASC antibodies at 4°C. After three washes with PBS, the cells were treated with secondary antibodies for 1 h at 25°C. Nuclei were stained for 10 min. Images were obtained using an ECLIPSE Ts2R/FL inverted microscope (Nikon, Japan).

### 2.13 Molecular docking

Molecular docking was conducted with the Glide module of Schrödinger (Schrödinger LLC, NY, USA) following a previously established method (Wang et al., 2025). The 3D structure of dehydrocostus lactone (DCL), 4-hydroxybenzoic acid (4-HBA), and gallic acid (GA) was drawn using ChemDraw software and subsequently optimized with the “LigPrep” module. The crystal structure of the human NLRP3 protein (PDB ID: 6NPY) was obtained from the RCSB Protein Data Bank (http://www.rcsb.org/pdb). The protein structure was processed using the Protein Preparation module, which included optimizing protein conformation, removing water molecules, minimizing energy, and adding any incomplete residues and hydrogen atoms. The pH value of the docking environment was set at 7.0 ± 0.5. While the binding site of ligands to NLRP3 was set as a grid box with dimensions 30 × 30 × 30 Å. All other parameters were set to their default values in Schrödinger.

### 2.14 Isothermal dose-response-cellular thermal shift assay (ITDR-CETSA)

To determine the binding between NLRP3 and active compounds, an ITDR-CETSA assay was performed (Tu et al., 2023). Briefly, LPS-primed THP-1 cells were lysed on ice for 30 min to extract proteins. The collected proteins (2.5 mg/mL) were incubated with DMSO or increasing concentrations of compounds for 1 h at room temperature. Later, the protein samples were heated at a single temperature point (56°C) for 4 min, after cooled at room temperature for 3 min, centrifuged at 15,000 rpm for 30 min to obtain soluble fractions, which were subsequently analyzed by Western blotting.

### 2.15 Animals

Thirty adult male Sprague-Dawley rats (250 g, 6–8 weeks old, specific pathogen-free grade) were procured from SPF Beijing Biotechnology Co., Ltd. (Animal Licence number SYXK 2023-0015, Beijing, China). The rats were contained in cages with a 12:12-h light-dark cycle, a temperature of 25°C ± 2°C, and a humidity of 55% ± 5%. Before the initiation of the experimental procedures, the rats were allowed a one-week acclimatization period, during which they had unrestricted access to diet and water. All protocols received approved and were adhered to the ethical guidelines of the Jiangxi Zhby Biotech Co., Ltd. Ethics Committee (No. LL-202405310001).

### 2.16 Establishment of MSU-induced acute gouty arthritis model

The rats were randomly assigned to five groups (n = 6): control, model (MSU), SWRL (400 mg/kg), SWRH (800 mg/kg), and colchicine (positive drug, 1 mg/kg). After 7 days acclimatization, the rats of the SWRL, SWRH, and colchicine groups were gavaged once daily for 6 days, whereas the rats of the remaining groups were gavaged an equivalent volume of 0.5% CMC-Na. One hour after drug administration on day 6, an AGA rat model was established by intra-articular injection of 0.2 mL of MSU suspension (25 mg/mL). Successful establishment of the model was confirmed by an observable contralateral protrusion in the joint cavity, along with redness and swelling of the joint. The hind paw volume of each group was measured at 0, 2, 4, 8, and 24 h following the MSU challenge using a toe volume-measuring instrument (KewBasis, China). According to Coderre’s method (Coderre and Wall, 1988), the gait of the rats was observed and scored as follows: grade 0, normal walking; 1 point, grade 1: slight lameness, the subject’s lower limbs were slightly bent, 2 points; grade 2: moderate lameness, the subject’s lower limbs just touched the ground, 3 points; grade 3: severe lameness, the subject’s lower limbs left the ground and walked on all three feet, 4 points. The joint inflammation index grading was as follows: grade 0 normal, 0 points; grade 1, mild swelling but bony signs with joint skin erythema, 2 points; grade 2, joint parts with limited swelling, bony signs disappeared, and joint erythema was obvious, 4 points; grade 3, joints outside the limbs with swelling, 6 points. Subsequently, all experimental groups were euthanized and tissues from the hind paws were homogenized. The supernatant obtained from the homogenate was collected for ELISA and Western blot analysis.

### 2.17 Histopathological study

Rat synovial tissue, liver, and right kidney were fixed with 4% paraformaldehyde for 48 h and then dehydrated with ethanol at a concentration gradient sequentially before being embedded in paraffin. These tissues were stained with hematoxylin and eosin (H&E) immediately after being sliced into 4 μm-thick sections. The pathological images of tissue sections were initially observed with an Olympus CKX53 inverted microscope (Tokyo, Japan) and then harvested by using the Pannoramic 250 digital slice scanner (3DHISTECH, Hungary) at a magnification of ×200.

### 2.18 Statistical analysis

All experimental analyses were conducted independently in triplicate. The *in vitro* data are presented as the mean ± standard deviation (SD), while the *in vivo* data are presented as the mean ± standard error of the mean (SEM) using GraphPad Prism 9.5 (San Diego, CA, USA). Significant differences among groups were assessed using a one-way analysis of variance (ANOVA). *p-*value <0.05 was considered statistically significant.

## 3 Results

### 3.1 Chemical profile of SWR by UPLC-MS analysis

**FIGURE 1:**
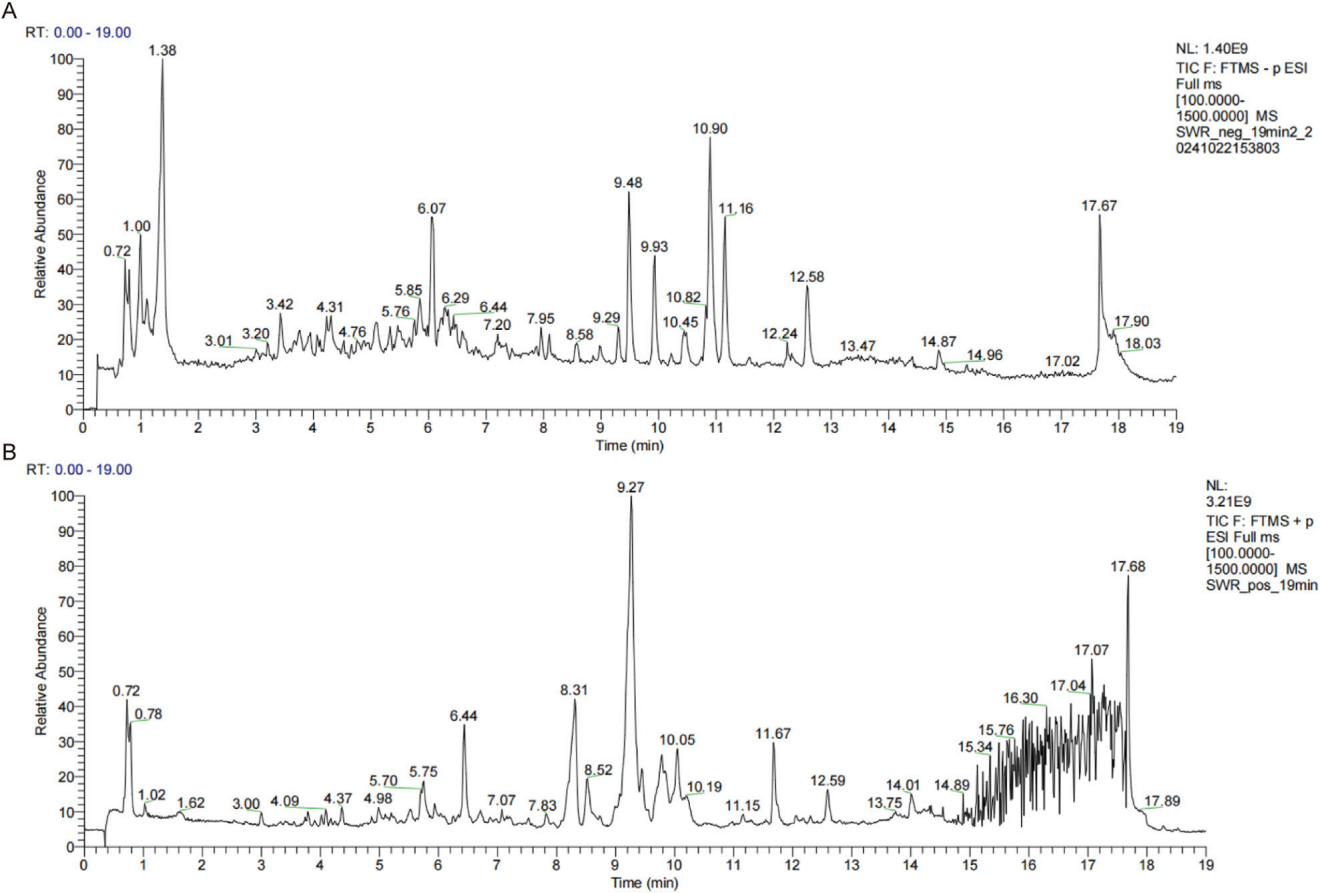
Chemical components analysis of SWR through UPLC-MS. **(A)** Total ion chromatogram obtained in negative ionization mode. **(B)** Total ion chromatogram obtained in positive ionization mode.

The chemical profile of SWR was performed by UPLC-MS, and the chromatograms of positive and negative ion modes were shown in Figure 1. The Xcalibur software was utilized to process and analyze MS data. After testing, 58 identified compounds were matched and applied for qualitative analysis, whose detailed results were shown in Table 2. These compounds were mainly polyphenols and terpenoids, which was consistent with previous study (Li Q. et al., 2022).

**TABLE 2:** Identified compounds in SWR obtained by UPLC-MS.

Entry	Rt (min)	Quasi-molecular ion	*m/z* measured	*m/z* calculated	Error (ppm)	Molecular formula	Compound identification
1	0.72	[M-H]^−^	180.0629	180.0634	2.69	C_6_H_12_O_6_	D-(+)-Galactose
2	0.72	[M+Na]^+^	193.0970	193.0950	10.28	C_7_H_15_NO_5_	(+)-valiolamine
3	1.00	[M-H]^−^	192.0627	192.0634	3.56	C_7_H_12_O_6_	D-(-)-Quinic acid
4	1.10	[M-H]^−^	129.0419	129.0426	5.32	C_5_H_7_NO_3_	4-Oxoproline
5	1.38	[M-H]^−^	170.0209	170.0215	3.65	C_7_H_6_O_5_	Gallic acid
6	1.62	[M+Na]^+^	150.0891	150.0892	0.71	C_6_H_14_O_4_	Triethylene glycol
7	1.76	[M+Na]^+^	126.0341	126.0317	19.11	C_6_H_6_O_3_	5-Hydroxymethylfurfural
8	2.54	[M+H]^+^	191.0940	191.0946	3.26	C_11_H_13_NO_2_	5-Methoxytryptophol
9	3.20	[M-H]^−^	138.0311	138.0317	4.29	C_7_H_6_O_3_	4-Hydroxybenzoic acid
10	3.42	[M-H]^−^	179.0578	179.0582	2.44	C_9_H_9_NO_3_	Hippuric acid
11	3.74	[M+H]^+^	355.1774	355.1784	2.68	C_21_H_25_NO_4_	Tetrahydropalmatine
12	3.76	[M-H]^−^	634.0818	634.0806	1.89	C_27_H_22_O_18_	Corilagin
13	3.78	[M+H]^+^	285.1356	285.1365	3.11	C_17_H_19_NO_3_	(R)-Coclaurine
14	4.09	[M+H]^+^	353.1254	353.1263	2.59	C_20_H_19_NO_5_	Protopine
15	4.31	[M-H]^−^	302.0068	302.0063	1.78	C_14_H_6_O_8_	Ellagic acid
16	4.37	[M+H]^+^	217.2035	217.2042	3.10	C_12_H_27_NO_2_	(2S,3R)-2-aminododecane-1,3-diol
17	4.51	[M-H]^−^	124.0518	124.0524	5.06	C_7_H_8_O_2_	4-Methylcatechol
18	4.76	[M-H]^−^	462.0805	462.0798	1.48	C_21_H_18_O_12_	Aureusidin 6-glucuronide
19	4.98	[M+H]^+^	211.0118	211.0126	3.57	C_9_H_9_NOS_2_	2-(1,3-Benzothiazol-2-ylsulfanyl)ethanol
20	5.33	[M-H]^−^	402.0954	402.0951	0.81	C_20_H_18_O_9_	Cerarvensin
21	5.52	[M+Na]^+^	227.1881	227.1885	1.86	C_13_H_25_NO_2_	Cyprodenate
22	5.70	[M+H]^+^	273.2658	273.2668	3.56	C_16_H_35_NO_2_	N-Lauryldiethanolamine
23	5.75	[M+H]^+^	317.2920	317.2930	3.11	C_18_H_39_NO_3_	2-Amino-1,3,4-octadecanetriol
24	5.76	[M-H]^−^	504.3458	504.3451	1.42	C_30_H_48_O_6_	Arjungenin
25	6.07	[M-H]^−^	594.1377	594.1373	0.62	C_30_H_26_O_13_	Maritimetin 6- (6″-p-coumarylglucoside)
26	6.44	[M+Na]^+^	414.2035	414.2042	1.77	C_24_H_30_O_6_	Bis(4-ethylbenzylidene)sorbitol
27	6.45	[M-H]^−^	284.0689	284.0685	1.51	C_16_H_12_O_5_	Glycitein
28	6.47	[M-H]^−^	460.2103	460.2097	1.28	C_25_H_32_O_8_	Prednisolone hemisuccinate
29	6.58	[M-H]^−^	158.1301	158.1307	3.66	C_9_H_18_O_2_	Nonanoic acid
30	6.97	[M+H]^+^	402.2371	402.2406	8.75	C_24_H_34_O_5_	Gamabufotalin
31	7.07	[M+H]^+^	230.1298	230.1307	3.82	C_15_H_18_O_2_	Dehydrocostus lactone
32	7.20	[M+H]^+^	282.1974	282.1984	3.42	C_20_H_26_O	dehydroretinaldehyde
33	7.52	[M+Na]^+^	380.2559	380.2563	0.97	C_22_H_36_O_5_	Limaprost
34	7.63	[M-H]^−^	298.2512	298.2508	1.37	C_18_H_34_O_3_	2-Oxooctadecanoicacid
35	7.95	[M-H]^−^	200.1772	200.1776	2.14	C_12_H_24_O_2_	Vulvic acid
36	8.10	[M-H]^−^	354.2200	354.2195	1.43	C_23_H_30_O_3_	Etretinate
37	8.31	[M+Na]^+^	514.3499	514.3506	1.29	C_28_H_50_O_8_	Trihexyl 2-(butyryloxy)-1,2,3-propanetricarboxylate
38	8.58	[M-H]^−^	470.3397	470.3396	0.20	C_30_H_46_O_4_	3α-Hydroxyglycyrrhetinate
39	8.97	[M-H]^−^	278.2250	278.2246	1.52	C_18_H_30_O_2_	α-Linolenic acid
40	9.27	[M+Na]^+^	396.2870	396.2876	1.44	C_23_H_40_O_5_	Pimilprost
41	9.29	[M-H]^−^	228.2088	228.2089	0.56	C_14_H_28_O_2_	Myristic acid
42	9.48	[M-H]^−^	340.2404	340.2402	0.51	C_23_H_32_O_2_	2,2′-Methylenebis (4-methyl-6-tert-butylphenol)
43	9.76	[M+H]^+^	432.2841	432.2876	8.03	C_26_H_40_O_5_	Latanoprost
44	9.93	[M-H]^−^	280.2403	280.2402	0.26	C_18_H_32_O_2_	Linoleic acid
45	10.05	[M+H]^+^	288.2444	288.2453	3.17	C_20_H_32_O	Taxa-4(20) 11(12)-dien-5α-ol
46	10.19	[M+Na]^+^	582.3328	582.3345	2.95	C_38_H_46_O_5_	1-[4-(3-{4-[(2S)-2,3-Dihydroxypropoxy]-3-methylphenyl}-3-pentanyl)-2-methylphenoxy]-3,3-dimethyl-4-(2-naphthyl)-2-butanone
47	10.24	[M-H]^−^	456.3608	456.3603	1.00	C_30_H_48_O_3_	Ursolic acid
48	10.45	[M-H]^−^	512.3500	512.3502	0.33	C_32_H_48_O_5_	Acetyl-11-keto-β-boswellic acid
	11.67	[M+H]^+^	512.3491		2.09		
49	10.82	[M-H]^−^	454.3448	454.3447	0.24	C_30_H_46_O_3_	β-Elemonic acid
	12.05	[M+H]^+^	454.3438		1.96		
50	10.90	[M-H]^−^	256.2403	256.2402	0.28	C_16_H_32_O_2_	Ethyl myristate
51	11.16	[M-H]^−^	282.2558	282.2559	0.28	C_18_H_34_O_2_	Oleic acid
52	11.57	[M-H]^−^	404.3138	404.3138	0.04	C_22_H_44_O_6_	Hexadecyl β-D-glucopyranoside
53	12.24	[M-H]^−^	498.3713	498.3709	0.79	C_32_H_50_O_4_	Tsugaric acid A
54	12.58	[M-H]^−^	284.2716	284.2715	0.25	C_18_H_36_O_2_	Octadecanoic acid
55	12.59	[M+H]^+^	283.2867	283.2875	2.86	C_18_H_37_NO	Stearamide
56	13.75	[M+H]^+^	323.3179	323.3188	2.81	C_21_H_41_NO	1-(14-methylhexadecanoyl)pyrrolidine
57	14.01	[M+H]^+^	311.3179	311.3188	2.92	C_20_H_41_NO	N,N-Dimethyloctadecanamide
58	15.63	[M-H]^−^	340.3342	340.3341	0.21	C_22_H_44_O_2_	Docosanoic acid

### 3.2 SWR inhibited NLRP3 inflammasome activation

**FIGURE 2:**
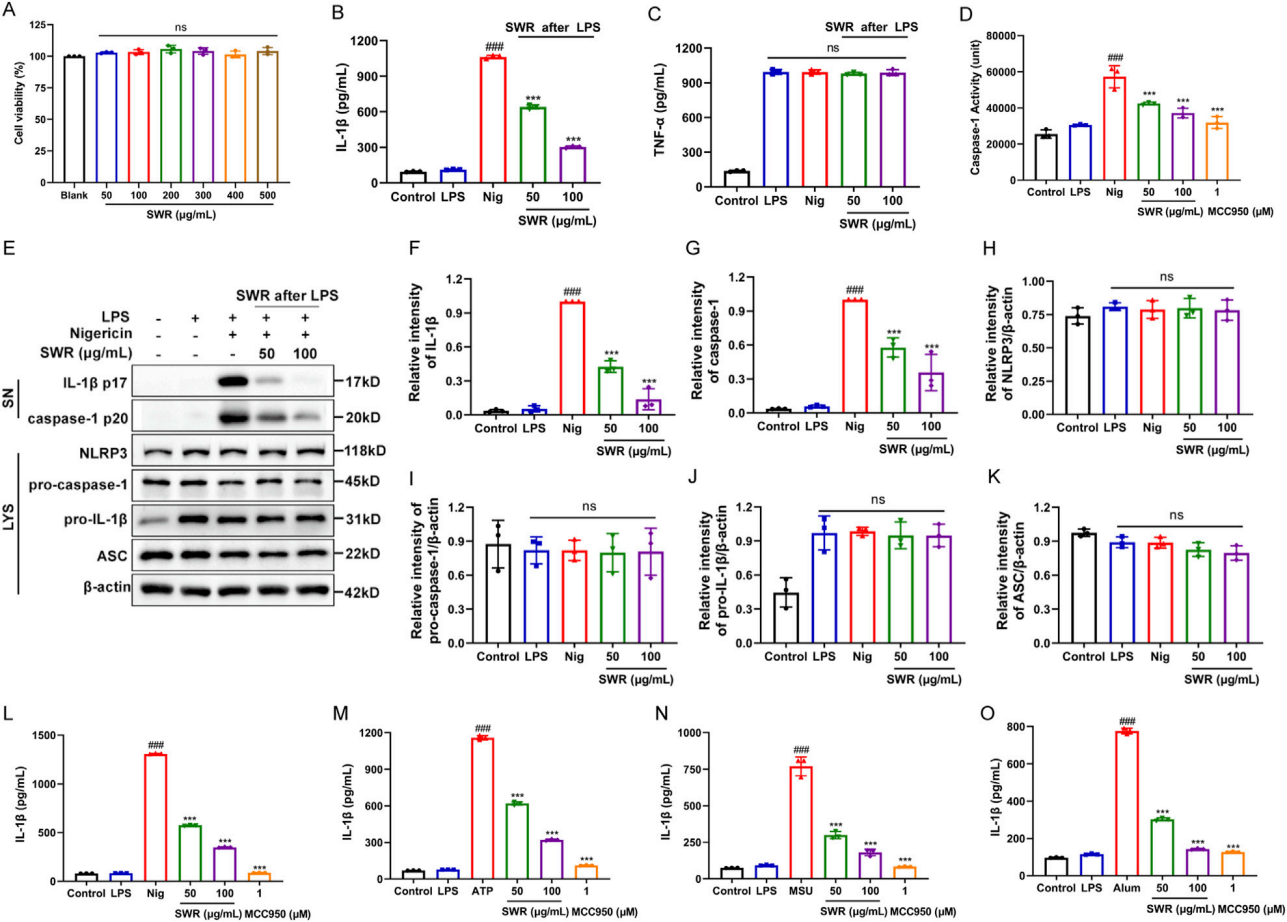
SWR inhibits NLRP3 inflammasome activation processes. **(A)** MTT was used to assess the cytotoxicity of THP-1 cells treated with different doses of SWR (50, 100, 200, 300, 400, 500 μg/mL) for 24 h. THP-1 cells were primed by LPS (1 μg/mL) for 3 h, and then treated with or without SWR in 50 and 100 μg/mL for 1 h, followed by the stimulation with 10 μM Nig for 40 min. IL-1β **(B)** or TNF-α **(C)** levels in supernatants were assessed using ELISA. **(D)** A caspase-1 activity assay kit was carried out to analyze activated caspase-1 activity in supernatants. **(E)** Western blot analysis of cleaved IL-1β and caspase-1 levels in culture supernatants (SN) and NLRP3, pro-IL-1β, pro-caspase-1, ASC, and β-actin in lysates (Input) of THP-1 cells. **(F–K)** The quantification of protein described in **(E)**. Different concentrations of SWR (50 and 100 μg/mL) were applied to LPS-primed THP-1 cells before being stimulated with nigericin **(L)**, ATP **(M)**, MSU **(N)**, Alum **(O)**, and ELISA was conducted to analyze IL-1β level in the supernatants. Data were expressed as means ± SD (n = 3). ^###^ *p* < 0.001 vs. control group; ***p < 0.001 vs. model group; ns, not significant (*p* > 0.05).

THP-1 cells were used to investigate the effects of SWR on NLRP3 inflammasome activation. The cell viability results revealed that SWR did not exhibit cytotoxicity at concentrations below 500 μg/mL (Figure 2A). While ELISA results indicated that IL-1β levels were elevated in the LPS plus Nig group relative to the control and LPS groups, confirming that the NLRP3 inflammasome activation model was successfully created. Next, we examined whether SWR (50 and 100 μg/mL) inhibited p20 cleavage and IL-1β secretion in THP-1 cells to verify the inhibitory effect of SWR on NLRP3 inflammasome activation. The results indicated that SWR dose-dependently suppressed IL-1β secretion without affecting TNF-α production (Figures 2B,C). In addition, SWR dose-dependently inhibited pro-caspase-1 (p45) form into p20, whereas intracellular pro-caspase-1 levels remained unchanged (p45) (Figures 2D–K). These findings meant that SWR inhibited NLRP3 inflammasome activation in THP-1 cells stimulated with LPS and Nig. Subsequently, we investigated whether SWR attenuated NLRP3 inflammasome activation triggered by other stimuli, including MSU, ATP, and Alum. As expected, SWR treatment effectively reduced the IL-1β maturation induced by MSU, ATP, and Alum in THP-1 cells (Figures 2L–O). Taken together, these observations suggest that SWR exhibits broad-spectrum inhibition of NLRP3 inflammasome activation.

### 3.3 SWR suppressed the cell pyroptosis induced by NLRP3 inflammasome activation

**FIGURE 3:**
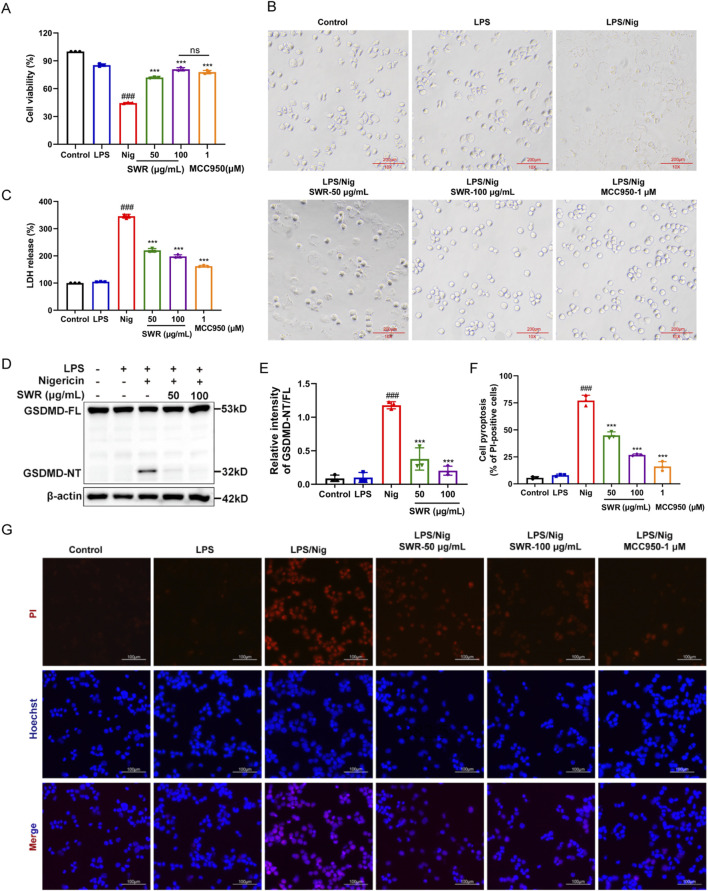
SWR suppresses pyroptosis induced by the activation of NLRP3 inflammasome. **(A)** Cell viability assay of THP-1 cells. **(B)** The observation of cell morphological changes. **(C)** LDH content in cell culture supernatant was detected by LDH assay. **(D)** THP-1 cells were treated with LPS in the presence of SWR (50 and 100 μg/mL) followed by nigericin. Cell lysates from THP-1 cells were immunoblotted for GSDMD and β-actin. **(E)** Quantification of cleaved-GSDMD described in **(D)**. **(F)** The percentage of pyroptosis cells in total cells was counted. **(G)** Cells were treated with PI/Hoechst 33342 and images were observed by fluorescence inverted microscope. Data were expressed as means ± SD (n = 3). ^###^ *p* < 0.001 vs. control group; ***p < 0.001 vs. model group; ns, not significant (*p* > 0.05).

NLRP3 inflammasome activation initiates caspase-1 activation, which subsequently cleaves the N-terminus of Gasdermin D (GSDMD-NT) to form plasma membrane pores, which in turn induces cell pyrolysis and LDH release, leading to cell pyroptosis (Kelley et al., 2019). Following SWR treatment, THP-1 cell viability increased in macrophages stimulated with LPS and Nig (Figure 3A). Cell morphology observation indicated that 100 μg/mL SWR could effectively prevent cell swelling and rupture caused by LPS plus Nig treatment (Figure 3B). While the measurement of LDH release demonstrated that SWR inhibited LDH release (Figure 3C). Additionally, SWR significantly decreased GSDMD-NT expression (Figures 3D,E). THP-1 cells stained with PI displayed red fluorescence. As shown in Figures 3F,G, SWR showed concentration-dependent suppression of red fluorescence production in THP-1 cells, and the inhibition in the SWR (100 μg/mL) group was similar to that of MCC950. These phenomena implied that SWR could inhibit pyroptosis induced by NLRP3 inflammasome activation.

### 3.4 SWR inhibited NLRP3 inflammasome priming processes

**FIGURE 4:**
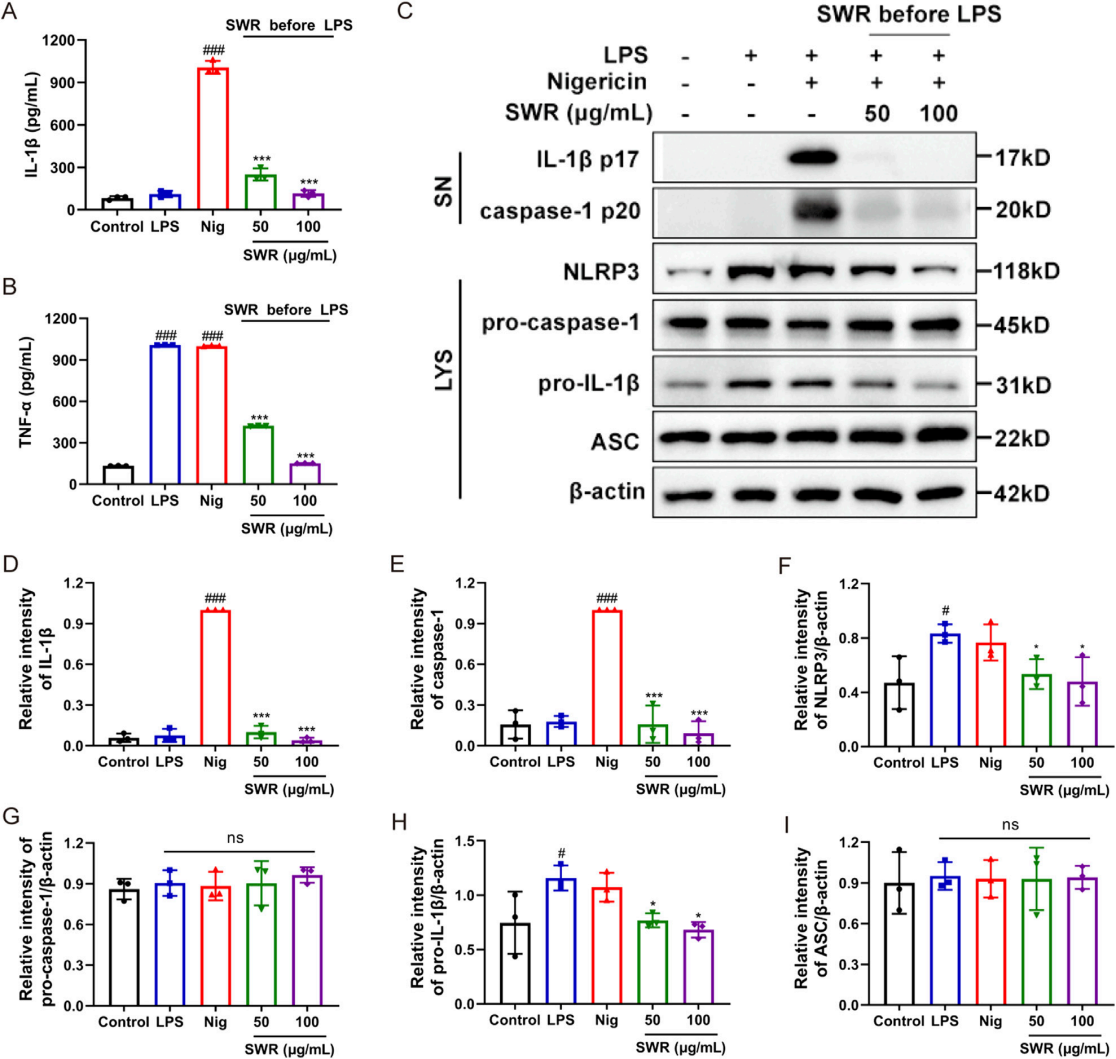
SWR inhibits NLRP3 inflammasome priming processes. SWR (50 and 100 μg/mL) was applied to THP-1 cells for 1 h, which was then primed with LPS (1 μg/mL) for 3 h, and Nig was finally used to stimulate the cells. ELISA was carried out to analyze IL-1β **(A)** and TNF-α **(B)** in supernatants. **(C)** Western blotting analysis of cleaved IL-1β and caspase-1 levels in culture supernatants (SN) and NLRP3, pro-IL-1β, pro-caspase-1, ASC and β-actin in lysates (Input) of THP-1 cells. **(D–I)** The quantification of protein described in **(C)**. Data were expressed as means ± SD (n = 3). ^#^ *p* < 0.05, ^###^ *p* < 0.001 vs. control group; *p < 0.05, ***p < 0.001 vs. model group; ns, not significant (*p* > 0.05).

In the process of NLRP3 inflammasome initiation, LPS stimulates the expression of NLRP3 and pro-IL-1β proteins through the TLR4/NF-κB pathway, which provides a sufficient protein environment for subsequent NLRP3 inflammasome assembly (Bauernfeind et al., 2009). Therefore, we investigated whether SWR influenced the LPS-induced priming stage of NLRP3 inflammasome activation. Given that TNF-α is a downstream product of the NF-κB signaling pathway and does not require NLRP3 inflammasome activation, the role of SWR in the priming process was examined by detecting the TNF-α, NLRP3, and pro-IL-1β levels. When THP-1 cells were treated with SWR before the LPS challenge, SWR significantly reduced the secretion of LPS-induced TNF-α (Figure 4B) and levels of pro-IL-1β and NLRP3 (Figures 4C–I). These findings meant that SWR suppressing NLRP3 inflammasome activation was related to the NF-κB signaling pathway, Most importantly, SWR inhibited the LPS-induced priming signal of NLRP3 inflammasome activation.

### 3.5 SWR inhibited NLRP3 inflammasome assembly

**FIGURE 5:**
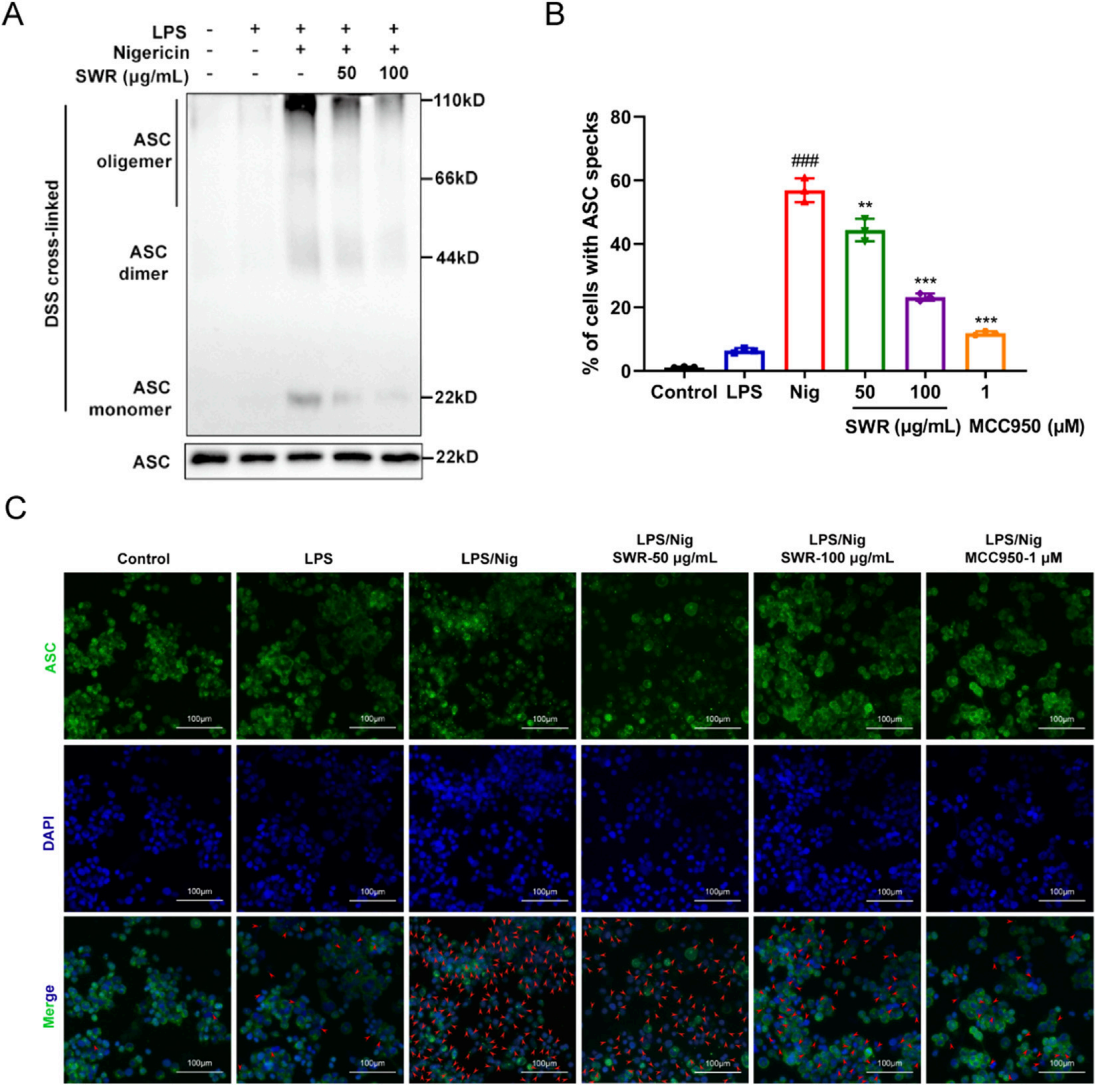
SWR inhibits ASC oligomerization. **(A)** Immunoblot analysis of ASC oligomerization in lysates derived from THP-1 cells treated with SWR (50 and 100 μg/mL). **(B)** The percentage of cells exhibiting ASC spots relative to the total cell count was quantified. **(C)** Representative immunofluorescence images illustrating ASC oligomerization in LPS-primed THP-1 cells with or without SWR (50 and 100 μg/mL) treatment for 1 h, followed by Nig stimulation for 40 min. Scale bar, 100 μm. Data were expressed as means ± SD (n = 3). ^###^ *p* < 0.001 vs. control group; **p < 0.01, ***p < 0.001 vs. model group.

ASC oligomerization, which participates in NLRP3 inflammasome assembly, is an essential step in the cleavage of caspase-1. Toward unraveling how SWR suppressed NLRP3 activation. The impact of SWR on ASC oligomerization is evaluated. The results revealed that pretreatment with SWR prior to Nig stimulation significantly reduced ASC oligomerization (Figure 5A), indicating that SWR interfered with the upstream signaling pathway of ASC oligomerization. Further immunofluorescence analysis clarified a reduction in ASC speck formation in THP-1 cells stimulated with LPS plus Nig after pretreatment with SWR, suggesting that SWR also inhibited ASC formation (Figures 5B,C). These facts pointed out that SWR inhibits NLRP3 inflammasome activation by blocking ASC oligomerization.

### 3.6 SWR inhibited MSU-induced acute gouty arthritis in rats

**FIGURE 6:**
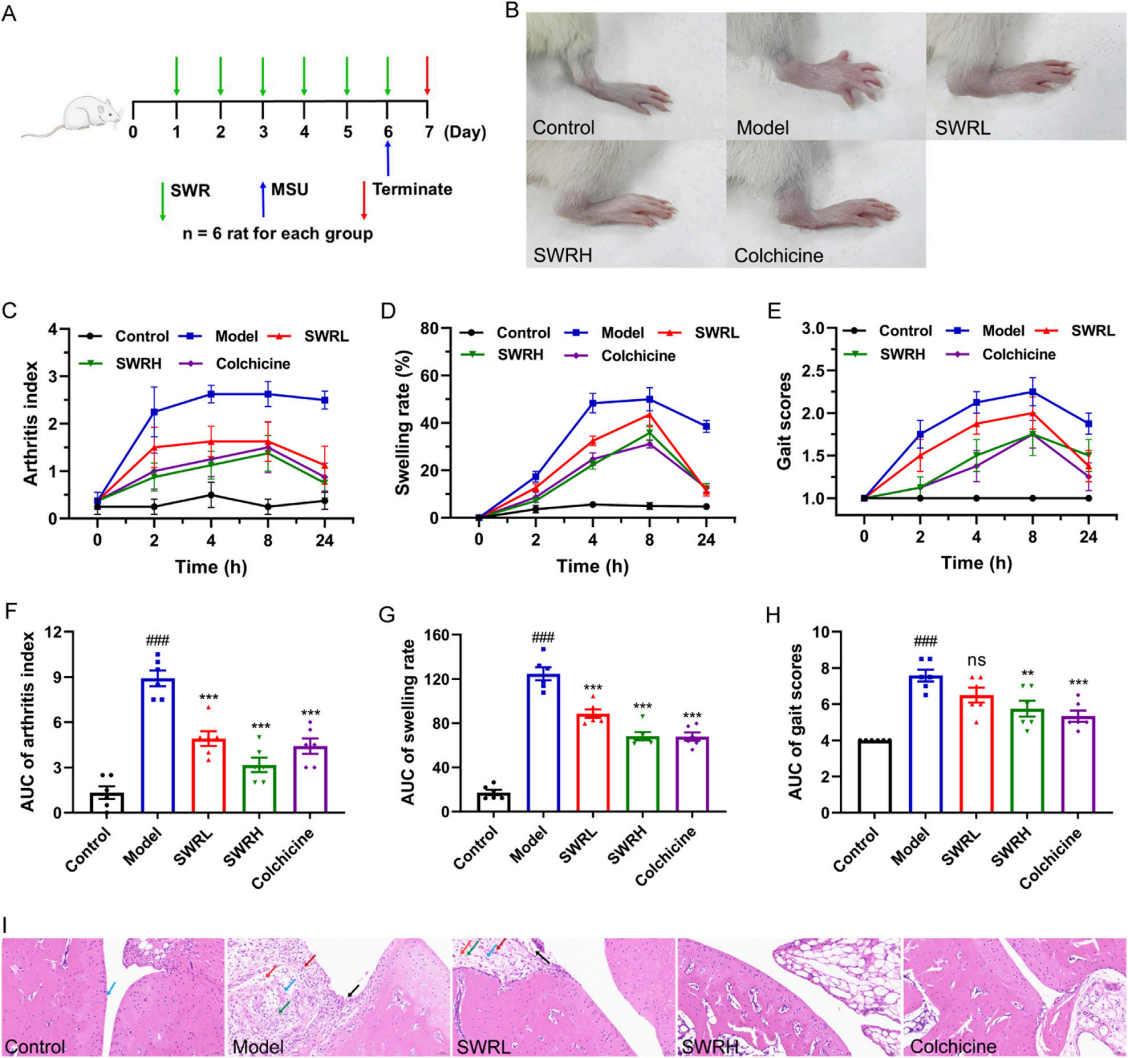
SWR inhibits MSU-induced ankle swelling in AGA rats. **(A)** Protocol of SWR treatment (400 and 800 mg/kg) in AGA rats. **(B)** Representative images of joint swelling. **(C,F)** Time course of changes of arthritis index. **(D,G)** Time course of changes of joint swelling. **(E,H)** Time course of changes of gait scores. **(I)** Histopathological study of ankle joints. Magnification, ×200; scale bars, 50 µm. Blue arrow, smooth cartilage surface; black arrow, necrosis and detachment of synoviocytes; green arrow, connective tissue hyperplasia; red arrow, inflammatory cell infiltration; dark red arrow, neovascularization. Data were expressed as means ± SEM (n = 6). ^*###*^ *p* < 0.001 vs. control group; **p < 0.01, ***p < 0.001 vs. model group; ns, not significant (*p* > 0.05).

Previous studies have revealed that the onset and progression of MSU-induced AGA are driven by the activation of the NLRP3 inflammasome (Szekanecz et al., 2019). In view of this, a rat model of AGA was established to evaluate the *in vivo* therapeutic efficacy of SWR. Rats were initially pretreated with SWR for 6 days, and the model was established by intra-articular injection of MSU 1 h after the final administration of SWR (Figure 6A). The doses of SWR (400 and 800 mg/kg) used in the experiments were triple and 1.5 times those clinically administered in adults. After MSU injection, the joints of the rats exhibited marked visible swelling (Figure 6B), representing that AGA was successfully induced. The joint volume in each group was measured using a toe volume-measuring instrument at 0, 2, 4, 8, and 24 h after MSU injection, and the degree of joint swelling was recorded. Compared to the model group, the joint swelling rate was significantly reduced in SWR-treated rats (Figures 6D,G). Encouragingly, the arthritis index was significantly increased in the model group of rats compared with that in the normal control group and significantly decreased after intervention with the positive drug colchicine and high-dose SWR (Figures 6C,F). In addition, the gait scores of rats in the model group were higher than those in the control group while the gait scores were significantly lower in the SWRH and colchicine groups than in the model group (Figures 6E,H). H&E staining images of the joint tissues showed that synovial cells within the knee joints exhibited varying degrees of necrosis, synovial tissue congestion, and inflammatory cell infiltration following MSU injection. Most importantly, inflammatory cell infiltration was markedly suppressed in the SWRH group (Figure 6I), demonstrating that SWR effectively alleviated AGA-related symptoms.

### 3.7 SWR inhibited NLRP3 inflammasome activation to prevent acute gouty arthritis in rats

**FIGURE 7:**
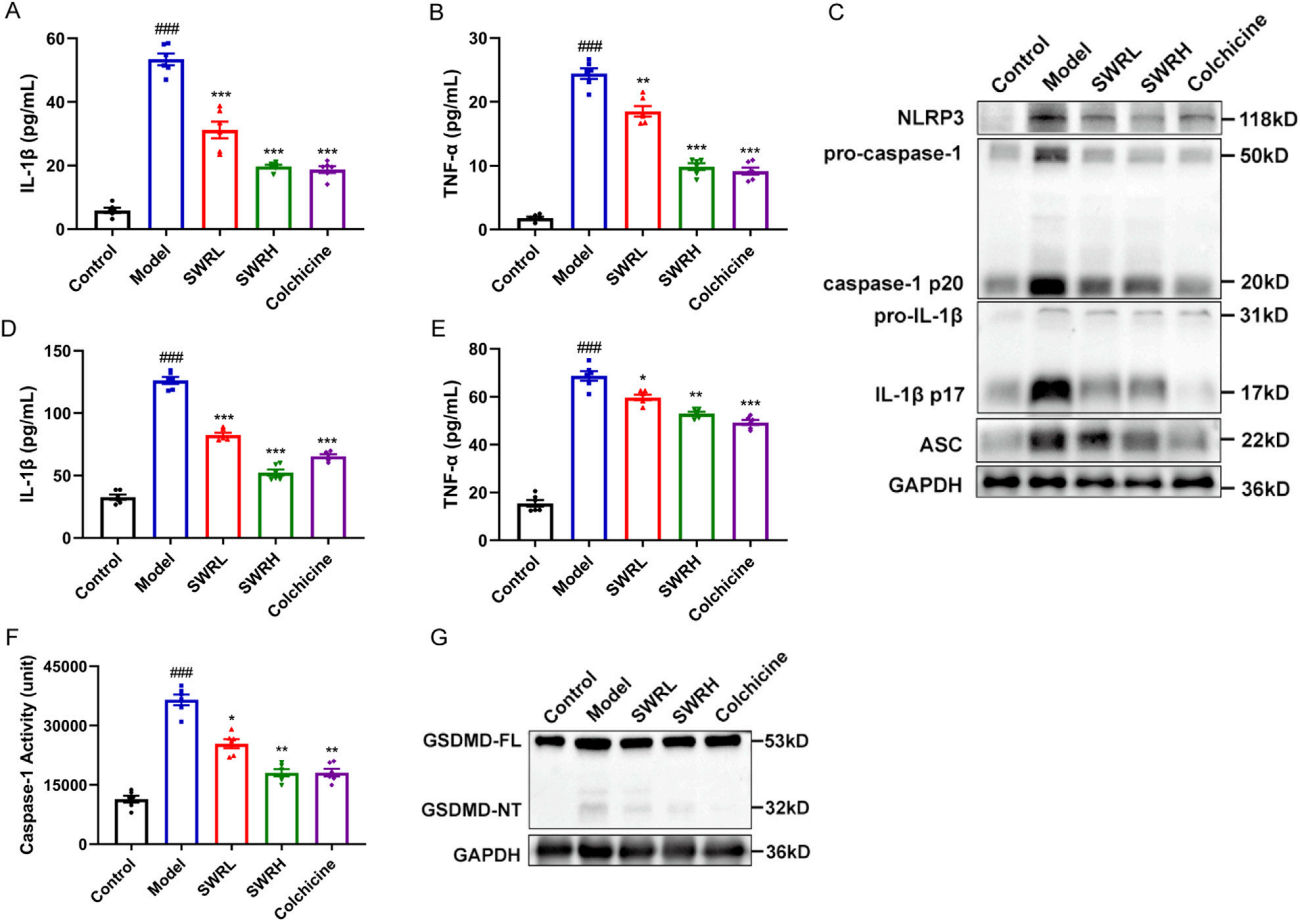
SWR inhibits NLRP3 inflammasome activation in AGA rats. ELISA analysis of IL-1β **(A)** and TNF-α **(B)** in AGA rat serum. **(C)** Western blot analysis of NLRP3, Caspase-1/pro-Caspase-1, IL-1β/pro-IL-1β, ASC, and GAPDH in the homogenate of paw tissue from rats. ELISA analysis of IL-1β **(D)** and TNF-α **(E)** in the homogenate of paw tissue. **(F)** Activity of caspase-1 in the homogenate of paw tissue. **(G)** Western blot analysis of GSDMD-NT/GSDMD-FL in the homogenate of paw tissue. Data were expressed as means ± SEM (n = 6). ^*###*^ *p* < 0.001 vs. control group; *p < 0.05, **p < 0.01, ***p < 0.001 vs. model group.

Next, emphasis is given to the relationship of the therapeutic effect of SWR on AGA with the inhibition of inflammasome activation. As shown in Figures 7A,B, compared to the model group, the production of IL-1β and TNF-α in rat serum in the SWR and colchicine groups were significantly reduced, a gradient decrease in the SWRL and SWRH groups can be observed, in which the SWRH group exhibited therapeutic efficacy equivalent to that of the colchicine group. The expression of inflammasome-related markers in the synovial tissues of the model group increased significantly, indicating successful modeling (Figures 7C,G). Interestingly, oral administration of SWRL and SWRH significantly downregulated the protein expression of NLRP3, caspase-1, IL-1β, ASC, and GSDMD. Furthermore, SWRL and SWRH decreased IL-1β (Figure 7D) and TNF-α levels (Figure 7E) and caspase-1 activity (Figure 7F) in synovial tissues of rats with AGA. These variations were consistent with the findings of the cellular experiments, depicting that SWR could attenuate AGA by inhibiting the NLRP3 inflammasome activation and reducing the production of IL-1β and TNF-α.

### 3.8 SWR displayed good safety in rats with AGA

**FIGURE 8:**
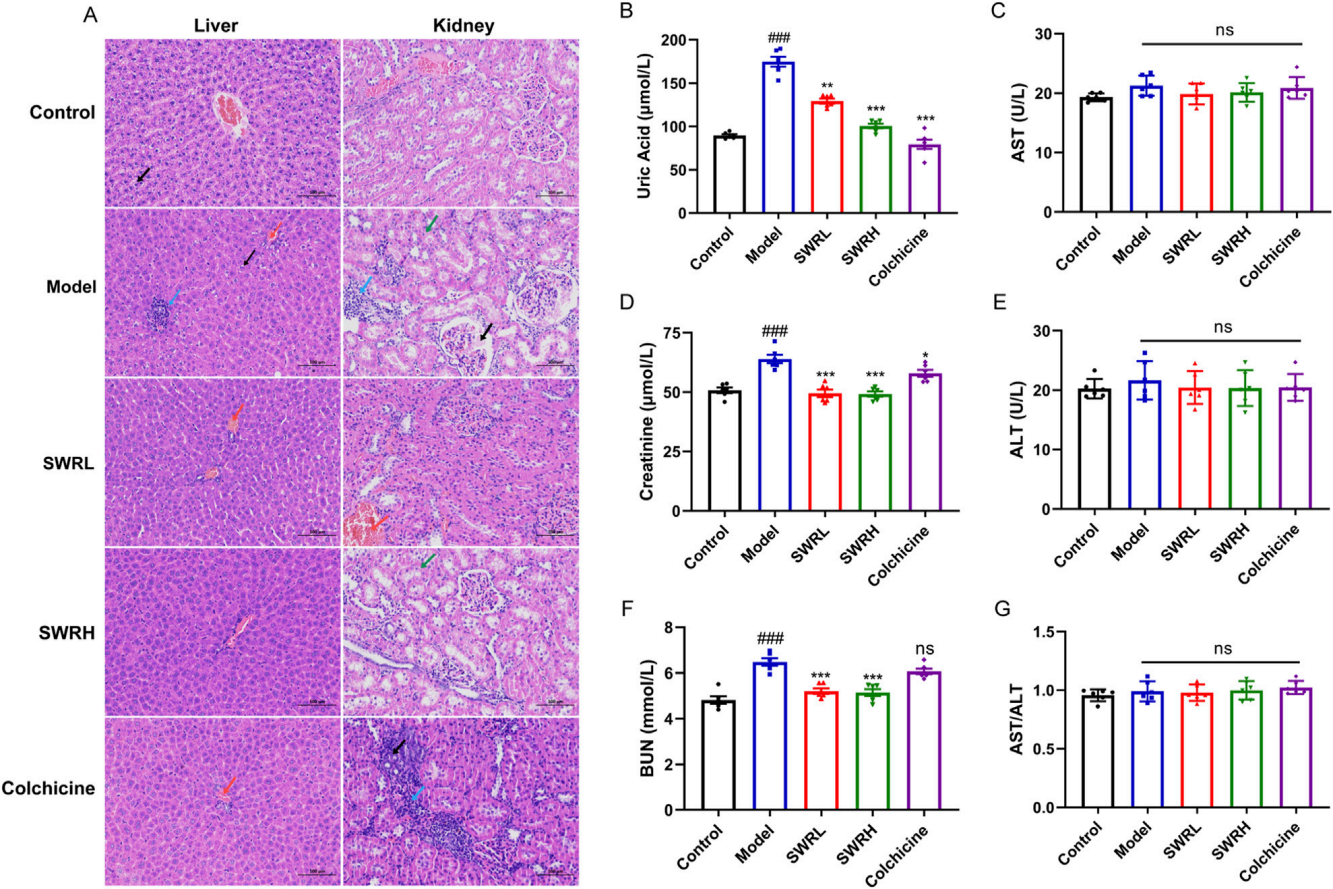
Effects of SWR on the liver and renal pathological changes in AGA rats. **(A)** Histopathological changes in the liver and kidney of rats in each group, with a magnification of ×200 and a scale bar of 100 µm. Black arrow, mild hepatocellular edema, and renal tubular atrophy; red arrow, venous stasis; blue arrow, lymphocyte infiltration; green arrow, hydropic degeneration of renal tubular epithelial cells. **(B)** Serum UA level. **(C)** Serum AST level. **(D)** Serum Cr level. **(E)** Serum ALT level. **(F)** Serum BUN level. **(G)** Serum AST/ALT ratio. The data are presented as means ± SEM (n = 6). ^*###*^ *p* < 0.001 vs. control group; *p < 0.05, **p < 0.01, ***p < 0.001 vs. model group; ns, not significant (*p* > 0.05).

As displayed by H&E staining images of livers (Figure 8A), the hepatocyte membranes, and cords in the control group exhibited integrity and were organized systematically and devoid of significant pathological alterations, meanwhile the hepatic sinusoids maintained a structurally normal appearance. Noticeably, compared to control groups, slight lymphocytic infiltration and a small number of hepatocytes with mild edema were observed in the model group. The pathology of the liver in the SWR group was similar to that of the control group, no significant change can be observed in AST/ALT ratios between the SWR groups and the control group, which suggested the safe hepatic profile of SWR (Figures 8A,C,E,G).

H&E staining images showed renal pathological changes in rats with AGA, in which slight hydropic degeneration of renal tubular epithelial cells and a small amount of lymphocyte infiltration appeared. Administration of colchicine does not alleviate renal damage. Inspiringly, both SWRL and SWRH alleviated histopathological damage in the kidney compared to that of the colchicine group (Figure 8A). The good renal protective effect of SWR can be derived from a marked reduction in serum creatinine (Cr) and blood urea nitrogen (BUN) concentrations of the SWR-treated group (Figures 8D,F). Simultaneously, the elevated level of serum uric acid (UA) in AGA rats was significantly decreased by SWR treatment. These observations implied dual anti-inflammatory and UA-lowering effects of SWR.

### 3.9 Identification of active compounds of SWR against NLRP3 inflammasome activation

**FIGURE 9:**
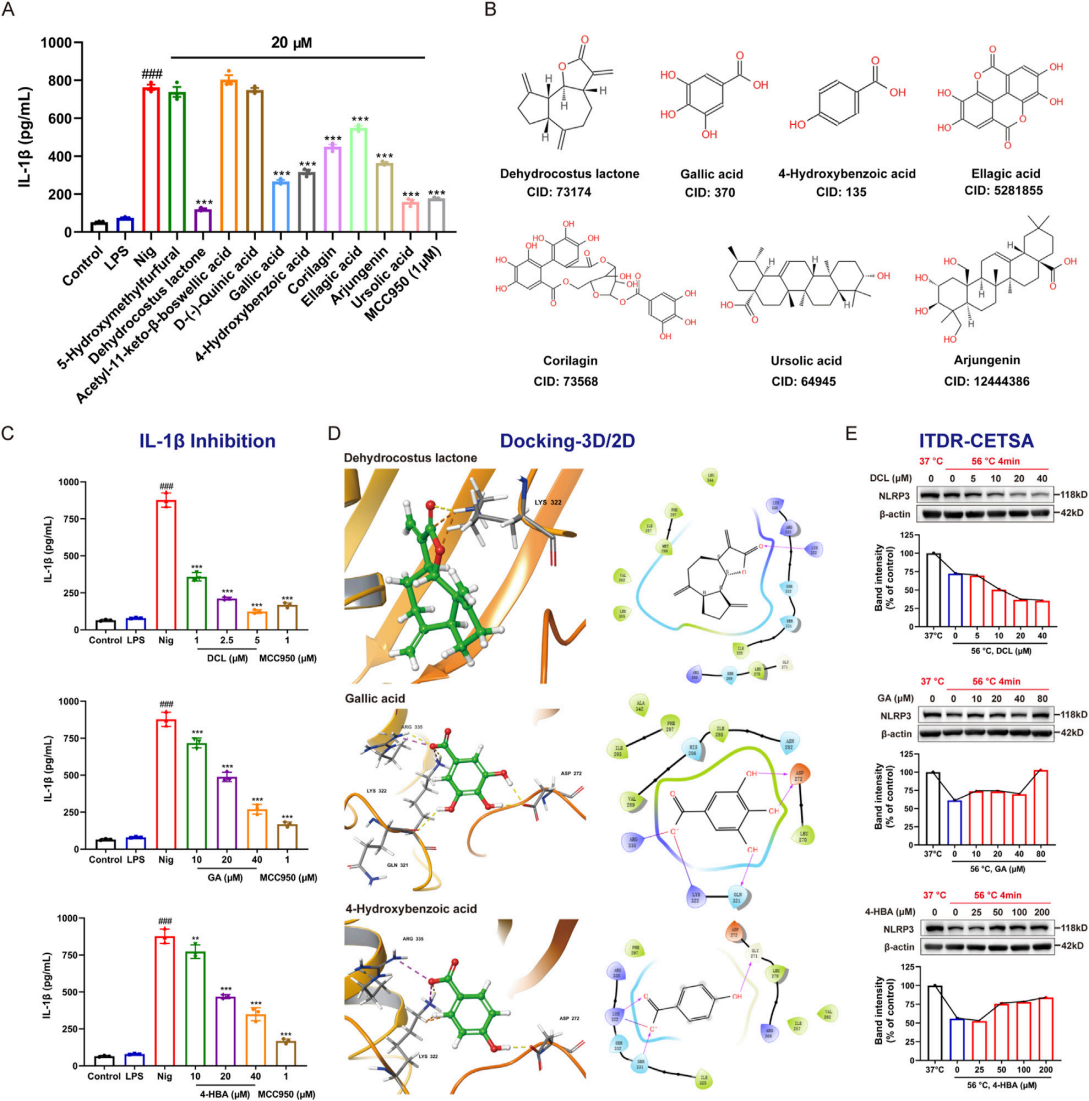
Active components of SWR against NLRP3 inflammasome activation **(A)** *In vitro* activity validation of ten compounds from SWR in THP-1 cells. **(B)** Structure and Pubchem CID of seven active compounds. **(C)** Inhibitory effects of three representative compounds on IL-1β production. **(D)** The docking analysis of three representative compounds with NLRP3 (PDB ID: 6NPY). 3D (left) interaction schematic: the hydrogen bonds in yellow, salt bridge in purple; 2D (right) interaction schematic: hydrogen bonds were shown as purple lines, salt bridge was in red-purple lines. **(E)** ITDR-CETSA analysis of three representative compounds. Data were expressed as means ± SD (n = 3). ^###^ *p* < 0.001 vs. control group; **p < 0.01, ***p < 0.001 vs. model group.

The above investigations has some interest findings, unfortunately, the active compounds of SWR against AGA remain unknown, particularly those related to the inhibition of NLRP3 inflammasome. The UPLC-MS analysis identified a total of 58 compounds, considering the commercial availability, ten compounds were selected and purchased for the subsequent bioactivity validation. Seven of them (Figure 9B), namely DCL, gallic acid (GA), 4-hydroxybenzoic acid (4-HBA), ursolic acid, arjungenin, corilagin, and ellagic acid, displayed good inhibitory activities on IL-1β production in LPS/Nig-induced THP-1 cells (Figure 9A). Further detailed evaluation of three potent compounds (DCL, GA, and 4-HBA) revealed their significant dose-dependent inhibition on IL-1β production, which disclosed they might possess good NLRP3 inflammasome inhibitory activity (Figure 9C). Molecular docking (Figure 9D) showed the strong interactions of three compounds with NLRP3 via hydrogen bonding. In brief, Lys322, ARG335, ASP272, GLN321, GLY271, and SER331 of NLRP3 protein are the key amino acids that interact with ligands. The carbonyl group of DCL, the free hydroxyl group of GA, and 4-HBA all form hydrogen bonds with the residue Lys322 of NLRP3. In addition, the presence of a salt bridge was observed in the binding mode of GA with NLRP3. These interaction modes indicated that the binding of three compounds to NLRP3 protein was quite stable. Subsequently, ITDR-CETSA analysis was adapted to determine whether the active compounds could directly bind to NLRP3 in the THP-1 cells. Transient heating at 56 °C than at 37 °C significantly caused NLRP3 protein denaturation, it is gratifying that incubation with GA and 4-HBA enhanced the resistance of NLRP3 to thermal denaturation. Additionally, incubation with DCL concentration-dependently reduced the thermal stability of NLRP3 (Figure 9E). These results indicate that GA, 4-HBA, and DCL can directly bind to NLRP3 in pathological environments, thereby inhibiting IL-1β production.

## 4 Discussion

AGA is considered a sterile autoimmune form of arthritis that involves rapid inflammation due to the MSU accumulating in the joints, marked by an abrupt onset of intense joint pain (Wang et al., 2024). Currently, treatment predominantly relies on pharmacological interventions including NSAIDs and colchicine. Notably, the unpleasant side effects cannot be ignored.

The NLRP3 inflammasome is a polyprotein composite primarily responsible for caspase-1 invasion, causing the release of proinflammatory cytokines and cell pyroptosis (Wang D. et al., 2020; Wang T. et al., 2023; Zou et al., 2023). Persistent and excessive activation of NLRP3 inflammasome can cause various inflammatory diseases such as Alzheimer’s disease and atherosclerosis (Daniels et al., 2016; Mangan et al., 2018). Recent studies have established an association between the NLRP3 inflammasome and the initiation and advancement of AGA (Lee et al., 2016; Szekanecz et al., 2019). Consequently, pharmacological agents targeting the inhibition of NLRP3 inflammasome have emerged as a potential therapeutic strategy for AGA (Zahid et al., 2019). Normally, the activation of NLRP3 inflammasome is divided into the priming phase and activation phase (Afonina et al., 2017). During the priming phase, PAMPs are detected by Toll-like receptors, resulting in the activation of the MAPK and NF-κB signaling pathways to upregulate NLRP3 and pro-IL-1β protein expression, of which LPS is a typical class of PAMPs (Zindel and Kubes, 2020). In the subsequent activation phase, signals, such as Nig and MSU, activate and assemble the NLRP3 inflammasome. Once NLRP3 inflammasomes are activated, pro-IL-1β is cleaved into the IL-1β form, expanding the inflammatory cascade in AGA (Jo et al., 2016).

Tibetan medicine displays positive clinical efficacy and low toxicity in preventing and treating AGA (Guo et al., 2022; Nima et al., 2024). SWR, a traditional Tibetan medicinal formulation, has been shown to be effective in the clinical management of AGA. It has recently been established that SWR could alleviate rheumatoid arthritis by restraining the MAPK and STAT3 signaling pathways, ameliorating HUA by regulating the MAPK and NF-κB signalling pathways (Li Q. et al., 2022; Xiong et al., 2023). It raised a question of whether SWR alleviated AGA via suppression of NLRP3 inflammasome activation. For these reasons, we embarked upon a systematic investigation of the anti-inflammatory effects of SWR on AGA rats and corresponding active compounds. Resulting observations unveiled that the suppression of NLRP3 inflammasome activation emerged as a critical mechanism contributing to the anti-AGA effects of SWR. SWR could both suppress NLRP3 inflammasome priming and activation processes and this inhibition is closely associated with its anti-inflammatory properties.

ASC is a crucial adaptor protein within the cytoplasm that connects pattern recognition receptors to pre-caspase-1. Upon triggering NLRP3 inflammasome sensors, ASC assembles into larger helical fibrils, forming ASC oligomers and specks that offer a molecular platform for autocatalytic cleavage and caspase-1 (Hoss et al., 2017). Thus, ASC oligomerization is essential for the activation of the NLRP3 inflammasome, and the regulation of ASC speckle formation is regarded as a promising approach for the treatment of inflammation. In this study, SWR suppressed ASC oligomerization, implying that it constrains NLRP3 inflammasome activation by obstructing inflammasome assembly. Unfortunately, the specific inhibition mechanism of ASC oligomerization remains unclear. More attention should be paid to this aspect.

It is widely known that AGA is an intense inflammation triggered by MSU sedimentation within the joints. MSU crystals are identified and phagocytosed by various intrinsic immune cells that enter the cell and activate intracellular signaling, including the formation of NLRP3 inflammasomes (Liu-Bryan, 2010). MSU crystals interact with intracellular NLRP3 inflammasomes and then secrete IL-1β into the extracellular compartment, resulting in neutrophil recruitment and the release of further inflammatory mediators, causing an amplified inflammatory cascade and accelerating the process of AGA (Torres et al., 2009). The above findings revealed that SWR effectively alleviated joint swelling and inflammatory pathological alterations in the synovial tissues of rats with AGA and the efficacy of the SWRL group was comparable to colchicine group. Meanwhile, SWR significantly suppressed caspase-1 activity and IL-1β production in the rat serum and paw tissue. Specifically, SWR decreased NLRP3, IL-1β, caspase-1, and ASC expression along with the cleavage of GSDMD-NT *in vivo*. These observations indicated that SWR alleviated MSU-induced AGA symptoms in rats through inhibiting NLRP3 inflammasome activation.

Colchicine, the first-line drug for AGA treatment, is highly effective but generated severe adverse effects (Terkeltaub, 2010). In clinical practice, the serum AST/ALT ratio reflects hepatic function (Chen et al., 2022), while renal function is assessed based on serum Cr and BUN (Patel et al., 2013; Zhong et al., 2022). No substantial liver damage and significant renal injury can be observed *in vivo* experiments. On the contrary, the colchicine group showed strong renal toxicity, whereas the SWRL and SWRH groups showed no hepatic injury and partial renal protection, as evidenced by the AST/ALT ratio, BUN and Cr levels, and histopathological results. Which confirms the favorable safety profile of SWR. HUA, characterized by an increased serum uric acid concentration, is considered a significant risk factor for AGA (Beyl et al., 2016; Mehmood et al., 2024). Approximately 5% of patients with HUA develop into AGA in a later period, experience significant pain, and have a diminished quality of life (Igel et al., 2017). So, in that sense, lowering serum UA levels may combat AGA. Notably, the significant serum UA-reducing effect of SWR was compatible with previous findings (Li Q. et al., 2022). It can be inferred that its alleviation of AGA was achieved by the dual anti-inflammatory and UA-lowering activities.

SWR is composed of ten materials, leading to its complex chemical profile. This complexity necessitates prompt identification of the active compounds responsible for its pharmacological effects. UPLC-MS analysis revealed 58 compounds, mainly polyphenols and terpenoids. Seven compounds (DCL, GA, 4-HBA, ursolic acid, arjungenin, corilagin, and ellagic acid) inhibited NLRP3 inflammasome activation. Among them, DCL was the most potent, corroborating previous findings (Chen et al., 2020). In addition, the inhibition of inflammasome activation of GA (Lin et al., 2020; Yu et al., 2023), 4-HBA, ursolic acid (Lei et al., 2023; Liu et al., 2024; Wang C. et al., 2020), corilagin (Li W. et al., 2022; Luo et al., 2022), and ellagic acid (Sun et al., 2021) has been reported previously. To our knowledge, this is the first report on the inhibitory activity of arjungenin (a common natural triterpenoid) against the NLRP3 inflammasome. Further molecular docking revealed that key hydrogen bonding interaction between NLRP3 and three active compounds (DCL, GA, and 4-HBA). Current findings and related reports demonstrate that phenolic are the main contributors in SWR to the amelioration of AGA by inhibiting the NLRP3 inflammasome activation.

It is worth noting that there are some limitations in this study. For example, due to the restriction of commercial availability, we only determined the bioactivities of 10 compounds that were available for purchase, and did not evaluate the activity of the remaining identified compounds. It will be necessary in the future to comprehensively evaluate the medicinal potential of SWR chemical components using virtual screening or artificial intelligence-driven drug screening methods. Some chemical components in SWR have been shown to suppress ROS production, including ursolic acid, 4-Hydroxybenzoic acid, corilagin and gallic acid, which might suggest that SWR can decrease ROS production (Kou et al., 2024; Lin et al., 2020; Liu et al., 2024; Luo et al., 2022). It is well-established that neutrophil extracellular traps (NETs) play a critical pathogenic role in gout by driving inflammation and tissue damage, which serves as an important biomarker and therapeutic target for gout and AGA (Liu et al., 2023; Remijsen et al., 2011; Schauer et al., 2014). Unfortunately, in this study, we mainly focused on its effect on NLRP3 inflammasome, with limited efforts on its impact on other pathological pathways of AGA. Future studies should switch to its regulation of ROS signaling and NETs, so as to provide a more comprehensive understanding of the anti-inflammatory mechanism of SWR.

**FIGURE 10:**
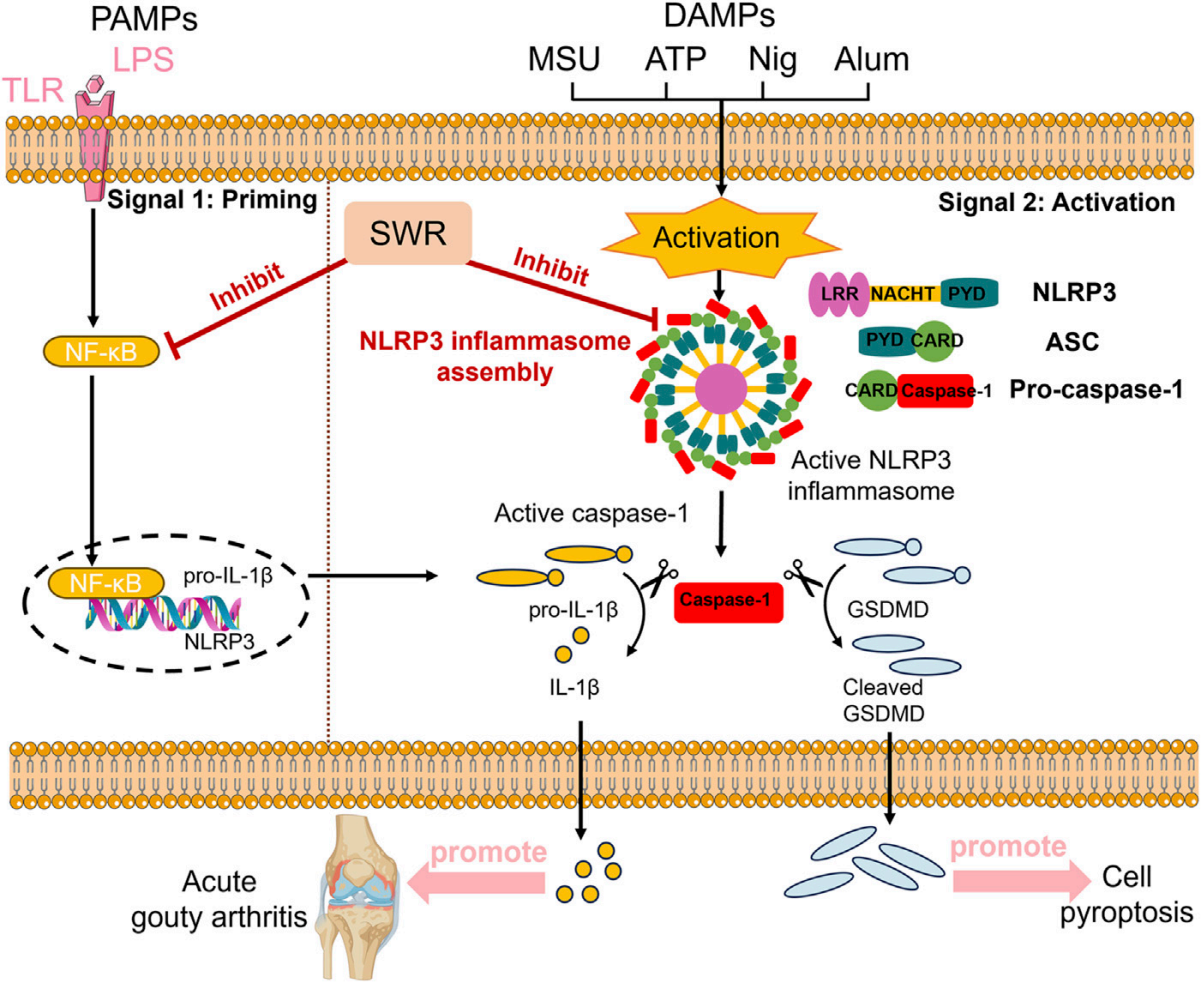
The mechanism scheme of SWR against AGA. SWR inhibits the release of pro-inflammatory cytokine and pyroptosis through inhibiting priming and activation processes of NLRP3 inflammasome, thereby attenuating acute gouty arthritis induced by MSU crystal.

In conclusion, current studies revealed that SWR significantly alleviated MSU-induced AGA symptoms in rats through dual UA-lowering and anti-inflammatory effects. The latter effect was primarily linked to its significant inhibition of NLRP3 inflammasome activation. The mechanism of action of SWR in treating AGA is detailed in Figure 10. SWR treatment effectively disrupted the inflammatory cascade initiated by the central inflammatory cytokine, IL-1β. Further investigation demonstrated that phenolics play key role in inhibition of inflammasome activation. This study offers a reasonable explanation for the effective and safe clinical use of SWR and provides valuable insights into its application.

## Data Availability

The raw Orbitrap data has been deposited to the MetaboLights repository, accession number MTBLS12617. Further inquiries can be directed to the corresponding authors.
