# Supplementary material for: Shi Wei Ru Xiang pill alleviates acute gouty arthritis through suppressing NLRP3 inflammasome activation
Source: Front Pharmacol. 2025 Jun 25;16:1595578. doi: 10.3389/fphar.2025.1595578 (PMC12237891; doi:10.3389/fphar.2025.1595578)
Supplement: Supplementary file 1 [file DataSheet1.docx]

**Supplementary data for original article**

**Shi Wei Ru Xiang pill alleviates acute gouty arthritis through suppressing NLRP3 inflammasome activation**

Na Wang ^a^, Puchen Zhao ^a^, Qin Yin ^a^, Lizi Li ^a^, Haiqi Xu ^a^, Can Yang ^a^, Yanbei Tu ^b,*^ and Yanfang Li ^a,*^

^a^ *School of Chemical Engineering, Sichuan University, Chengdu, Sichuan 610065, China*

^b^ *School of Pharmacy,* *Jiangsu University, Zhenjiang, Jiangsu 212013, China*

*Corresponding authors at: Yanbei Tu, School of Pharmacy, Jiangsu University, Zhenjiang, Jiangsu 212013, China; Yanfang Li, School of Chemical Engineering, Sichuan University, Chengdu, Sichuan 610065, China.

*E-mail address:* [yanbeitu@ujs.edu.cn](mailto:yanbeitu@ujs.edu.cn) (Y. Tu), [lyf471@vip.163.com](mailto:lyf471@vip.163.com) (Y. Li).

| **No.** | **Content** | **Page** |
| --- | --- | --- |
| 1 | **Abbreviations** | 1 |
| 2 | **Fig. S1.** Raw data of western blots for Fig. 1E (n=3). | 2 |
| 3 | **Fig. S2.** Raw data of western blots for Fig. 2D (n=3). | 3 |
| 4 | **Fig. S3.** Raw data of western blots for Fig. 3C (n=3). | 4 |
| 5 | **Fig. S4.** Raw data of western blots for Fig. 4A (n=3). | 5 |
| 6 | **Fig. S5.** Raw data of western blots for Fig. 6C and Fig. 6G (n=6). | 6 |

**Abbreviations**

AGA, acute gouty arthritis; MSU, monosodium urate; NLRP3, NOD-like receptor thermal protein domain associated protein 3, the nucleotide-oligomerization domain-like receptor (NLR) family pyrin domain-containing 3; ASC, adaptor protein apoptosis-associated speck-like protein containing a CARD; Caspase-1, cysteinyl aspartate specific proteinase-1; DAMPs, damage-associated molecular patterns; PAMPs, pathogen-associated molecular patterns; IL-1β, interleukin-1β; IL-18, interleukin-18; IBD, inflammatory bowel disease; NSAIDs, nonsteroidal anti-inflammatory drugs; TFP, Twenty-Five Wei'er Tea Pills; SWR, Shi Wei Ru Xiang Pill; MAPK, mitogen-activated protein kinase; STAT3, signal transducer and activator of transcription 3; NF-κB, nuclear factor kappaB; HUA, hyperuricemia; THP-1 cells, tohoku hospital pediatrics-1 cells; LPS, lipopolysaccharide; Nig, nigericin; UPLC-MS, ultra performance liquid chromatography-mass spectrometry; FBS, fetal bovine serum; PMA, phorbol 12-myristate 13-acetate; Alum, aluminum salts; ATP, adenosine triphosphate; MTT, thiazolyl blue; DMSO, dimethyl sulfoxide; SWRL, treatment group with a low dosage of SWR; SWRH, treatment group with a high dosage of SWR; CMC-Na, sodium carboxymethyl cellulose; ELISA, enzyme-linked immunosorbent assay; TNF-α, tumor necrosis factor-α; PI, propidium iodide; LDH, lactate dehydrogenase; TCA, trichloroacetic acid; WB, western blotting; PMSF, phenylmethylsulfonyl fluoride; BCA, bicinchoninic acid; SDS-PAGE, sodium dodecyl sulfate-polyacrylamide gel electrophoresis; PVDF, polyvinylidene fluoride; TBST, tris-buffered saline Tween-20. HRP, horseradish peroxidase; ECL, enhanced chemiluminescence; BSA, bovine serum albumin; DAPI, 4',6-diamidino-2-phenylindole; DSS, disuccinimidyl suberate; NP-40, Nonidet P-40; H&E, hematoxylin and eosin; DCL, dehydrocostus lactone; 4-HBA, 4-hydroxybenzoic acid; GA, gallic acid; ITDR, isothermal dose-response; CETSA, cellular thermal shift assay; ANOVA, analysis of variance; SD, Standard Deviation; SEM, standard error of mean; GSDMD, gasdermin D; ALT, alanine amino transferase; AST, aspartate aminotransferase; BUN, blood urea nitrogen; UA, uric acid; Cr, creatinine; TLRs, toll-like receptors; NACHT, nucleotide binding and oligomerization domain.

**Raw data of western blots**

**
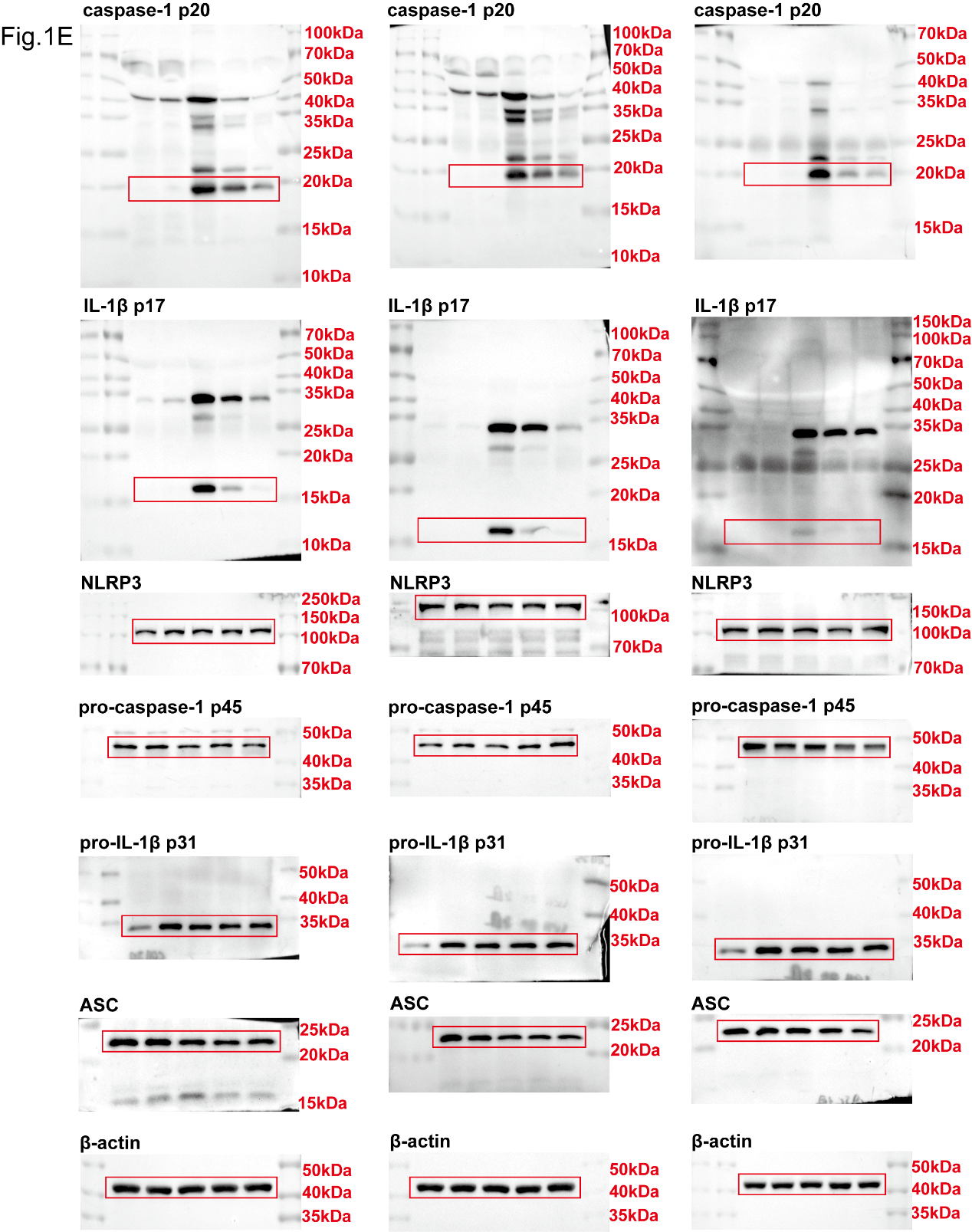
**

**Fig. S1.** Raw data of western blots for Fig. 1E (n=3).

**
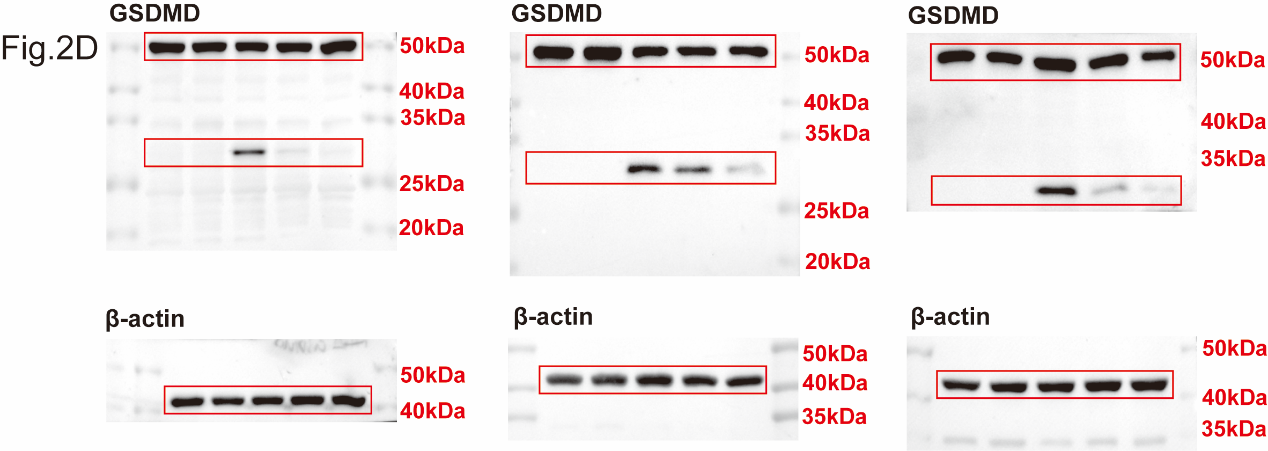
**

**Fig. S2.** Raw data of western blots for Fig. 2D (n=3).

**
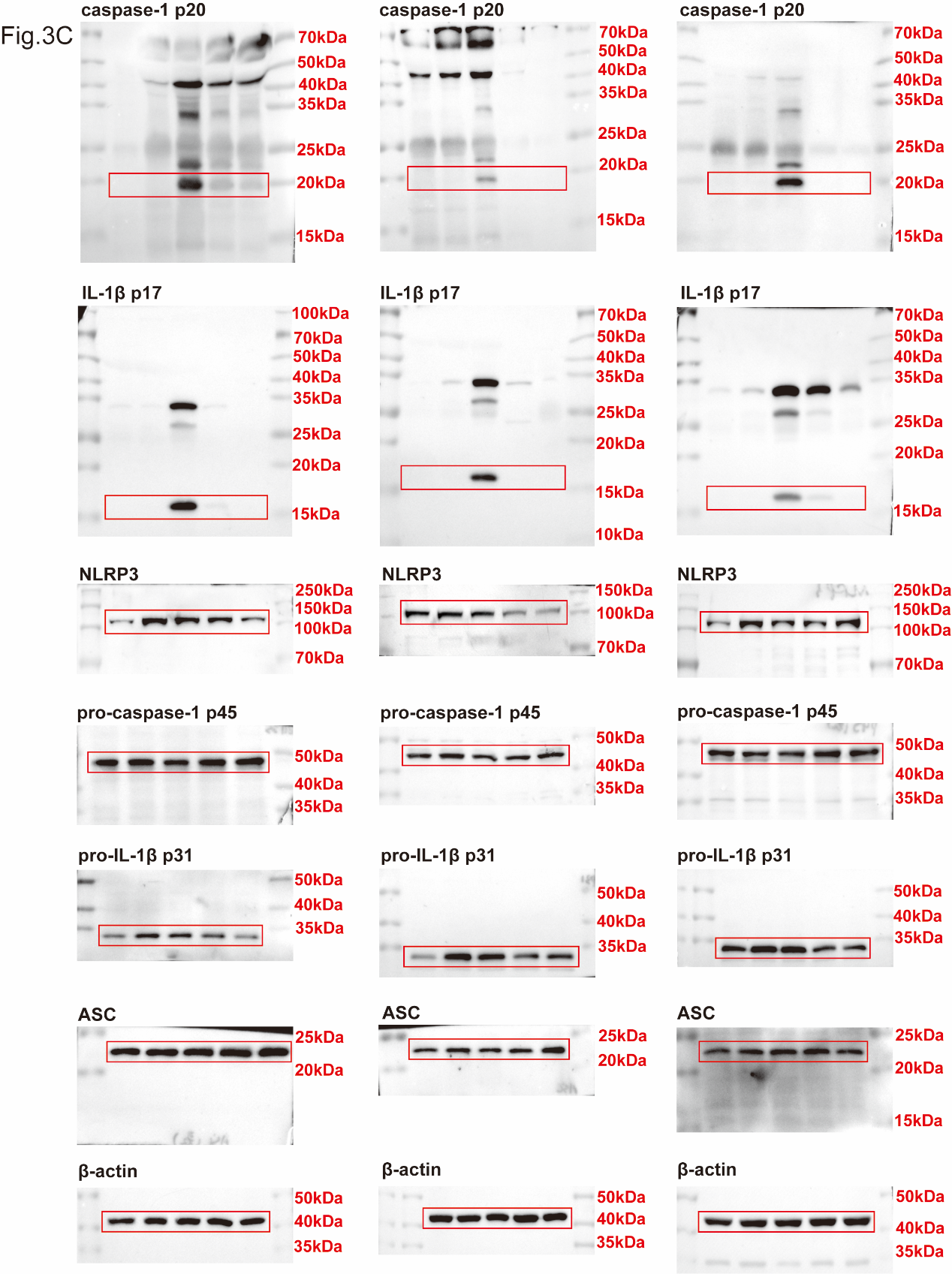
**

**Fig. S3.** Raw data of western blots for Fig. 3C (n=3).


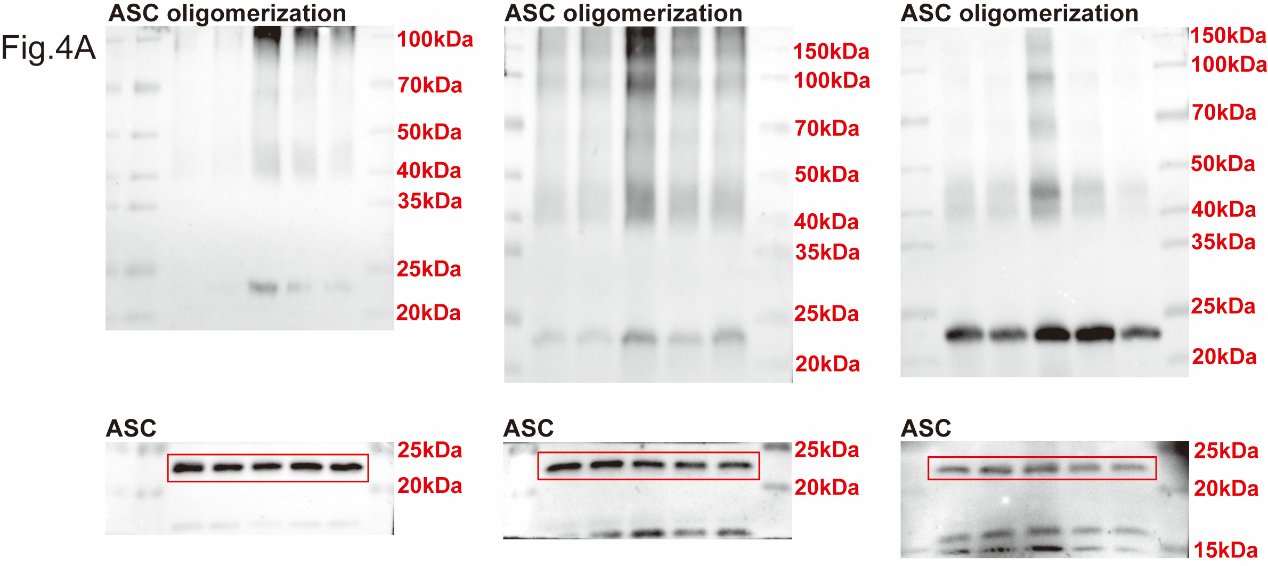


**Fig. S4.** Raw data of western blots for Fig. 4A (n=3).


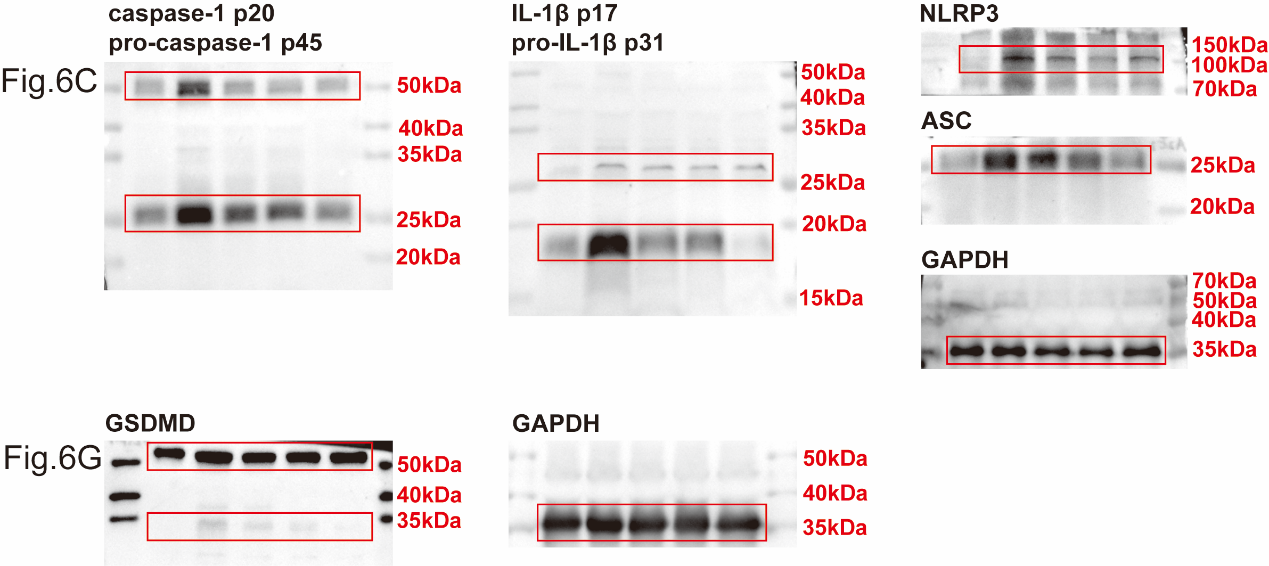


**Fig. S5.** Raw data of western blots for Fig. 6C and Fig. 6G (n=6).
